# Building a eukaryotic chromosome arm by de novo design and synthesis

**DOI:** 10.1038/s41467-023-43531-5

**Published:** 2023-11-30

**Authors:** Shuangying Jiang, Zhouqing Luo, Jie Wu, Kang Yu, Shijun Zhao, Zelin Cai, Wenfei Yu, Hui Wang, Li Cheng, Zhenzhen Liang, Hui Gao, Marco Monti, Daniel Schindler, Linsen Huang, Cheng Zeng, Weimin Zhang, Chun Zhou, Yuanwei Tang, Tianyi Li, Yingxin Ma, Yizhi Cai, Jef D. Boeke, Qiao Zhao, Junbiao Dai

**Affiliations:** 1 CAS Key Laboratory of Quantitative Engineering Biology, Guangdong Provincial Key Laboratory of Synthetic Genomics and Shenzhen Key Laboratory of Synthetic Genomics, Shenzhen Institute of Synthetic Biology, Shenzhen Institute of Advanced Technology, Chinese Academy of Sciences, Shenzhen, China; 2 State Key Laboratory of Cellular Stress Biology, Innovation Center for Cell Signaling Network, School of Life Sciences, Faculty of Medicine and Life Sciences, Xiamen University, Xiamen, Fujian 361102 China; 3 University of Chinese Academy of Sciences, Beijing, China; 4 Institute of Molecular Physiology, Shenzhen Bay Laboratory, Shenzhen, China; 5 Manchester Institute of Biotechnology, University of Manchester, Manchester, M1 7DN UK; 6 Institute for Systems Genetics and Department of Biochemistry and Molecular Pharmacology, NYU Langone Health, New York, NY 10016 USA; 7 Department of Biomedical Engineering, NYU Tandon School of Engineering, Brooklyn, NY 11201 USA; 8 Shenzhen Branch, Guangdong Laboratory for Lingnan Modern Agriculture, Key Laboratory of Synthetic Biology, Ministry of Agriculture and Rural Affairs, Agricultural Genomics Institute at Shenzhen, Chinese Academy of Agricultural Sciences, Shenzhen, China

**Keywords:** Genome, Synthetic biology, Chromosomes

## Abstract

The genome of an organism is inherited from its ancestor and continues to evolve over time, however, the extent to which the current version could be altered remains unknown. To probe the genome plasticity of *Saccharomyces cerevisiae*, here we replace the native left arm of chromosome XII (*chrXIIL*) with a linear artificial chromosome harboring small sets of reconstructed genes. We find that as few as 12 genes are sufficient for cell viability, whereas 25 genes are required to recover the partial fitness defects observed in the 12-gene strain. Next, we demonstrate that these genes can be reconstructed individually using synthetic regulatory sequences and recoded open-reading frames with a “one-amino-acid-one-codon” strategy to remain functional. Finally, a synthetic neochromsome with the reconstructed genes is assembled which could substitute *chrXIIL* for viability. Together, our work not only highlights the high plasticity of yeast genome, but also illustrates the possibility of making functional eukaryotic chromosomes from entirely artificial sequences.

## Abstract (web summary)

In *Saccharomyces cerevisiae*, the left arm of chromosome XII only requires 12 genes to maintain cell viability, whereas 25 genes are needed for robust fitness. Here the authors demonstrate that the entire arm can be replaced by a neochromosome with completely artificial sequences.

## Introduction

*Mycoplasma genitalium* was previously regarded as the bacterium with the smallest genome that can grow in axenic culture^1,2^, however, in 2016, the record was broken after the creation of JCVI- syn3.0 with a genome only 531 kbp in size, reduced from over 1.0 Mbp in the original *Mycoplasma mycoides*^3^. In this genome, only 473 genes, including the set of essential and quasi-essential genes, were retained^3,4^. For the model bacterium *Escherichia coli*, it has a genome at 4.6 Mbp, encoding 4434 genes. Through rounds of deletion, this was reduced by up to 15%, constituting the loss of 743 genes, including mobile DNAs^5^. The reduced genome strain was reported to not only preserve good growth profiles and protein production, but to also have beneficial properties including high electroporation efficiency and stability of recombinant plasmids.

Compared to prokaryotes, studies on genome reduction in eukaryotes are very limited. The genome of *Saccharomyces cerevisiae (S. cerevisiae)*, a model eukaryotic organism, is 12 Mbp in length and encodes about 6000 genes^6^. Using chromosome-splitting and loss techniques, it has been reported that 5% reduction of the *S. cerevisiae* genome could improve the productivity of ethanol and glycerol^7^. Since 2006, a group of researchers have committed to the construction of a designer version of the yeast genome (known as Sc2.0), in which the yeast genome size will be reduced about 8% through deletion of the retrotransposon related sequences, introns and subtelomeric repeat sequences^8^. In the Sc2.0 genome, numerous loxPsym sites were introduced to facilitate Synthetic Chromosome Rearrangement and Modification by LoxPsym-mediated Evolution (SCRaMbLE)^9^. Upon the activation of SCRaMbLE, large scale genome rearrangements such as inversion, deletion and duplication were observed and strains with particular phenotypes including resistance to higher temperature, for example, were identified^10^. To facilitate genome reduction, we recently developed an iterative SCRaMbLE-based genome compaction strategy, which allowed us to remove about 40% of *synXIIL* while the cells remained viable at 30 °C in rich medium^11^. However, how much more DNA could be further removed from this chromosome arm without impairing cell viability remains elusive.

Besides the content, regulation of gene expression by non-coding sequences adds another layer of complexity, particularly in eukaryotic organisms. Many functional elements such as enhancers, transcription factor binding sites, the TATA box, the transcription start site and poly(A) sites reside within these sequences even though these cis regulatory sequences are not systematically defined for every gene. Recently, synthetic promoters and terminators have been designed based on regulatory sequences with varied activity^12–15^. One challenge in synthetic genomics is to understand regulatory networks to such an extent that it is possible to design artificial sequences that effectively restore their function. Additionally, due to codon degeneracy and usage bias, different nucleotide sequences for the same amino acids can have substantially different functional impacts^16^. Up to now, genome-wide codon compression has only been successfully applied in *E. coli*^17–19^. Permissive and restrictive synonymous recoding schemes are largely unexplored in eukaryotes. Recently, using several essential genes as tested, we employed a codon compression scheme, in which only one codon is used for each amino acid, and swapped the regulatory sequences with those of *CYC1*^20^. Notably, we found that 7 out of the 10 reconstructed genes could complement their deletion from the haploid genome. However, whether cells can tolerate a chromosome or even an entire genome with such dramatical sequence modifications remains unknown.

The left arm of *chrXII* in *S. cerevisiae* (*chrXIIL*) is 150,827 bp long, ranking as the seventh shortest yeast arm. It contains 74 genes (62 protein coding genes, 9 dubious genes, 2 pseudogenes, 1 tRNA coding gene), 3 autonomously replicating sequences and 1 Ty1 LTR (Saccharomyces Genome Database, https://www.yeastgenome.org/). Among these genes, 10 are defined as essential based on the phenotype of individual knockout mutations and their ability to support spore viability in a heterozygous diploid. The length of proteins encoded by genes on *chrXIIL*, showed a similar distribution pattern to that of genome-wide genes (Supplementary Fig. 1a). The average number of directly evidenced gene ontology (GO) terms per gene on *chrXIIL* was slightly higher than most of the other chromosome arms, suggesting higher functional complexity to be considered during reconstruction (Supplementary Fig. 1b). To expand the understanding of genome plasticity in eukaryotes, we used this chromosome arm to explore the maximum extent of changes that can be made without affecting basic functions. A neochromosome was initially designed to facilitate the relocation of essential genes that are dispersed throughout *chrXIIL*. Using a partially synthetic chromosome XII, we systematically probed sequence essentiality in *chrXIIL* by targeted DNA deletion, chromosome truncation and gradual gene replenishment. Eventually, a series of neochromosomes, capable of substituting for *chrXIIL* for cell viability were constructed.

## Design of a neochromosome as a flexible carrier of exogenous DNA

**Fig. 1:**
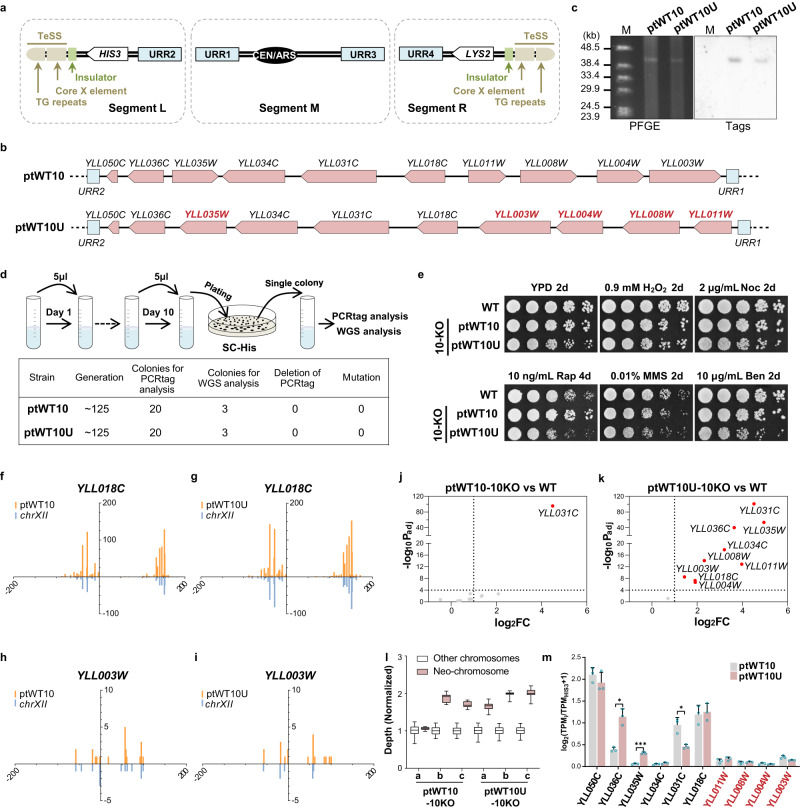
A designer neochromosome to harbor essential genes from the left arm of *chrXII*. **a**, The design of neochromosome. Marker genes, white; TeSS, brown; Insulators, green; URRs, blue. **b** Arrangements of the ten essential genes (pink) in ptWT10 and ptWT10U. Arrows point to the direction of transcription. The genes with changed transcription direction on ptWT10U are labeled in red. **c** PFGE and southern blotting analysis of the assembled neochromosomes. M: λ DNA-Mono Cut Mix. Source data are provided as a Source Data file. Images are representative of at least three independent experiments. **d** Stability test of neochromosomes. BY4742 cells containing ptWT10 or ptWT10U were cultured in SC-His medium for about 125 generations before PCR analysis and whole genome sequencing. **e** Fitness analysis of strains on different growth conditions. 10-fold gradient dilutions were conducted for cells in this study. WT: BY4741. H_2_O_2_: hydrogen peroxide; Noc: nocodazole; MMS: methyl methanesulfonate; Rap: rapamycin; Ben: Benomyl. **f–i** Full-length transcriptome analysis of BY4742 with ptWT10 or ptWT10U neochromosome. The transcripts on neochromosome (orange) and native transcripts (*chrXIIL*, blue) were mirrored. On the x-axis, negative number represents distance upstream of ATG and positive values indicate distance downstream of the stop codon. The Y-axis represents the counts of detected transcripts. **j**, **k** Transcription analysis of the ten essential genes in ptWT10-10KO or ptWT10U-10KO using DESeq2 to calculate Benjamini-Hochberg adjusted *p* values and fold change values. WT: BY4742. Differential expression in this study was defined as |log_2_FC| > 1 and -log_10_ (Adjusted *p*-value) >4. FC, fold change. Dashed lines, the threshold defined above. Red, differentially expressed essential genes on neochromosomes. **l** The copy number of neochromosomes in ptWT10-10KO or ptWT10U-10KO. a,b,c are three independent biological repeats. The mean read-depth of native chromosomes (*n* = 11,845 1 kb-sized bins) was normalized to 1. For the neochromosomes, *n* = 36 1 kb-sized bins. The bounds of the box were the upper and lower quartile with the median value in the center. The whiskers indicated 5th and 95th percentile. Source data are provided as a Source Data file. **m** Normalized expression of genes on ptWT10 (*n* = 3) and ptWT10U (*n* = 3). Expression of each gene was normalized to *HIS3* on neochromosomes. The data are presented as the mean and SD. A two-tail unpaired Welch’s t-test were employed to calculate the *p* values, which were adjusted by Benjamini-Hochberg method. *P < 0.05, **P < 0.01, ***P < 0.001. Source data are provided as a Source Data file. The exact *p* value was listed in Source data.

Linear artificial chromosomes containing structural elements for segregation, replication and stability (i.e., centromere, autonomous replication sequences and telomeres) have been reported to behave like native chromosomes^21,22^. However, de novo construction of a linear chromosome with capability for future applications remains limited. To produce a flexible carrier of exogenous DNA, we designed a neochromosome with unique features allowing it to be assembled easily in vivo. The overall construct design is shown in Fig. 1a.

The telomere seed sequences (TeSS), that is, the telomeric TG repeat plus the Core X element, which have been constructed and functional tested previously^8^, served as the telomeres. To ensure replication and segregation of the neochromosome, the centromere sequence from Chromosome II and an origin of replication (ARS208) were placed in the middle. It is well known that genes placed near the telomeres will be silenced—a phenotype called the telomere position effect (TPE)^23^. Therefore, to reduce TPE, a short insulator sequence (10 tandem repeats of TTAGGG) was put adjacent to TeSS to block the spreading of heterochromatin^24^. In addition, to allow robust integration of exogenous DNA with high specificity, two 500 bp random sequences, designated as the universal recombination regions (URRs)^25^, were also included in each chromosomal arm. Finally, two auxotrophic markers, *HIS3* and *LYS2* were inserted into the left and right arm respectively, to facilitate the selection of the assembled neochromosome. The structural elements of the neochromosome were divided into three parts, segments L, M, and R, which could be assembled together in yeast by homologous recombination.

## Construction of neochromosomes carrying essential genes from *chrXIIL*

We used the designed neochromosome to carry the 10 known essential genes of *chrXIIL*. We positioned these genes in two different formats (Fig. 1b): 1) in the same relative chromosomal position and transcriptional orientation as on the native chromosome (designated as ptWT10), and 2) unifying the transcriptional direction to point towards the telomere (ptWT10U). In ptWT10, the sequences and directions of essential genes were the same as those in a circular eArray reported to effectively compensate for the loss of a single native gene^11^. In ptWT10U, four genes *YLL003W*, *YLL004W*, *YLL008W*, *YLL011W* were flipped together, leading to a change of their relative position (Fig. 1b). In addition, since *YLL035W* and *YLL036C* share a bi-directional promoter and the intergenic region is unable to sustain the function of *YLL036C*^20^, we chose the reported *CYC1* promoter to achieve functional expression of both genes and maintain consistent regulation in ptWT10U. The sequences of ptWT10 and ptWT10U are listed in Supplementary Data 1.

To construct the neochromosomes, the three structural fragments, i.e., fragment L, M, and R, together with PCR-amplified essential-gene fragments were co-transformed into BY4742 (Supplementary Fig. 2a). Subsequently, cells containing assembled chromosomes were selected in a medium lacking histidine and lysine, randomly isolated and confirmed by PCR (Supplementary Fig. 2b–e). Next, the strains were analyzed by Pulsed-field gel electrophoresis (PFGE) followed by Southern Blotting. As shown in Fig. 1c, a band of around 40 kb was detected in both PFGE and Southern Blotting, indicating the successful construction of the neochromosome. Finally, nanopore sequencing was performed which revealed that both ptWT10 and ptWT10U are present in the cells with sequences as designed (Supplementary Fig. 2f, g). However, we observed several single nucleotide substitutions and insertions in the neochromosome (Supplementary Data 1) which do not affect the function of each essential gene (see below), and therefore, no further correction was performed. Interestingly, we found that the telomeric TG repeats, in both neochromosomes, significantly expanded (Supplementary Fig. 2h, i), indicating the TeSS end grew a new telomere successfully^8,26^.

Since the neochromosomes are relatively short (about 40 kb) and contain loxPsym sites in segments L and R, we firstly tested if such sequences remain stable in cells as reported^27^. As shown in Fig. 1d, we inoculated the strains in selective liquid medium and cultured the cells by 1000-fold dilution every day for ten consecutive days, accounting for about 125 mitotic generations. The cells were analyzed by both PCR analysis and next generation sequencing. For three independent clones tested, we found that none of them had changes in PCRtags or nucleotide sequences. These results demonstrate that the linear neochromosomes are stable in the cells.

## Essential genes relocated to the neochromosome could substitute their native counterparts for viability

To test whether genes relocated to linear neochromosomes function equivalently to those in native loci, we constructed strains with ptWT10 or ptWT10U in which the original genomic copies of these essential genes were deleted sequentially (designated ptWT10-10KO and ptWT10U-10KO). ptWT10-10KO showed indistinguishable growth from that of wild type under various conditions (Fig. 1e). This indicates that ptWT10 functions well to replace the native genes. ptWT10U-10KO cells exhibited a slight growth defect in rich medium (Fig. 1e). These results suggest that the general function of these essential genes is largely unaffected after direct relocation to the neochromosome, whilst changes in regulatory sequences or the orientation of transcription seem to result in fitness defects.

Since we rearranged the position of these essential genes by placing them side by side, especially in ptWT10U, it might lead to interference between adjacent transcription units (TUs)^28^. Therefore, we examined whether there were any abnormalities in transcription initiation and termination sites of each gene in the two neochromosomes using the isoform-sequencing (Iso-seq) method, a high-throughput method to identify all full-length transcripts within a cell. From the Iso-seq results of BY4742 containing either ptWT10 or ptWT10U, the full-length transcripts of nine essential genes on the neochromosomes, were identified using specific PCRTags (except for *YLL050C*, which is too short to contain PCRTags)^29^. For genes with the same transcription direction, such as *YLL018C*, the transcription start sites (TSS) and termination sites (TES) on the neochromosomes are similar to those in the native transcriptome (Fig. 1f, g, Supplementary Fig. 3a, b). The genes that were flipped on the neochromosomes, such as *YLL003W*, they were also transcribed similarly to native transcriptome (Fig. 1h, i, Supplementary Fig. 3c–e). For *YLL035W* and *YLL036C*, the TES and TSS showed a similar pattern between ptWT10 and the native genome locus, while the promoter and terminator of *CYC1* used in ptWT10U changed the transcription pattern as expected (Supplementary Fig. 3f–h). In addition, we did not observe any abnormal transcripts that extended through two adjacent genes (Supplementary Fig. 3i). These results indicated that the sequences we used for each gene were sufficient to regulate the transcription initiation and termination processes.

To examine whether the transcription level of genes was altered when relocated to the neochromosome, the expression of the ten genes in ptWT10-10KO and ptWT10U-10KO was analyzed by RNA-seq (Fig. 1j, k). Consistent with the above Iso-seq results, the transcripts of most genes identified on both ptWT10 and ptWT10U showed clear boundaries between different TUs (Supplementary Fig. 4a). We found that in ptWT10-10KO, all 10 essential genes except for *YLL031C* were transcribed at similar levels to their genomic counterparts in BY4742. Unexpectedly, 9 out of the 10 essential genes, including the two with altered transcription regulatory sequences, were overexpressed in ptWT10U-10KO (Fig. 1k and Supplementary Data 2). Besides, more other differentially expressed genes were identified in ptWT10U-10KO than ptWT10-10KO (190 vs 118, Supplementary Fig. 4b). ptWT10U-10KO also showed a more obvious transcriptome-wide perturbation than ptWT10-10KO when compared to BY4742 (Supplementary Fig. 4c).

## The neochromosomes exist in cells with variable copy number

The elevated expression of essential genes in ptWT10U-10KO lead us to ask whether the copy number of neochromosome had changed. Similar to the method for DNA copy number estimation using read-depth of the next-generation sequencing data^30^, we evaluated long-read sequencing data and calculated the ratio of the mean depth of the neochromosome to that of all native chromosomes as the average copy number of the neochromosome per haploid genome. As shown in Fig. 1l, we found that the neochromosomes exist in cells in one to two copies.

To eliminate the effects of DNA copy number, we normalized the expression level of each gene on the neochromosome to that of *HIS3*, which is located on the left arms of the neochromosomes. As shown in Fig. 1m, three genes, including the two with changed promoters and *YLL031C*, showed obviously different transcription levels between ptWT10 and ptWT10U, while none of the four “flipped” native genes, namely *YLL011W*, *YLL008W*, *YLL004W*, and *YLL003W*, showed significantly different transcription levels. These results suggest that the regulatory sequences, rather than the orientation of transcription, plays important roles in local gene regulation.

## Only 12 genes are sufficient to replace *chrXIIL* for viability

Previously, we found over half of the nonessential genes in *synXIIL* could be deleted by SCRaMbLE^11^. However, further compaction attempts using the same method failed, which presumably may be partially due to the extremely slow growth of the final strain ZLY349^11^. To probe the minimal gene set to support cell viability, two additional strategies were carried out here.

At first, we systematically examined the essentiality of sequences in the left arm of *chrXII* using CRISPR/Cas9 technology. The six regions flanking essential genes, which were either partially deleted or retained in ZLY349^11^, were knocked out individually (Supplementary Fig. 5a). Notably, all deletions generated viable strains, despite the growth defects exhibited by two of the generated strains (Supplementary Fig. 5b). These results suggested that all these non-essential regions could be removed.

**Fig. 2:**
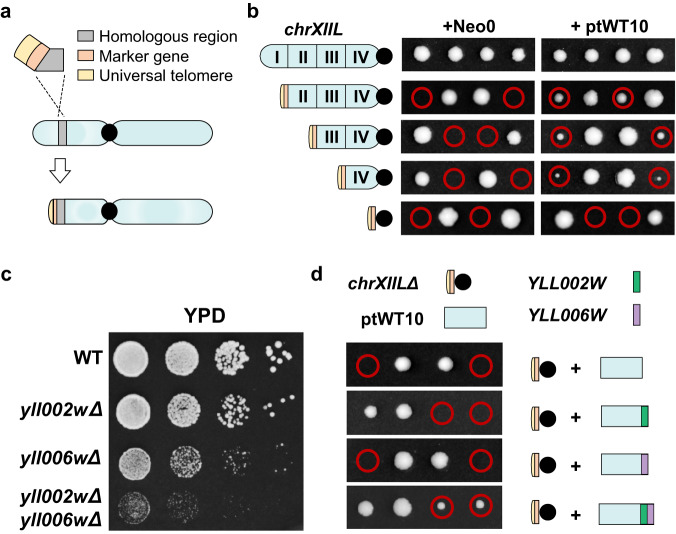
Only 12 genes are sufficient to replace *chrXIIL* for viability. **a** Schematic diagram of the method for chromosomal truncation. **b** Tetrad analysis of heterozygous diploid strains with different *chrXIIL* truncations. **c** Fitness tests of strains carrying *yll002w* and *yll006w* deletion. WT: BY4742. **d** Tetrad analysis of heterozygous diploid strains with one *chrXIIL* removed. *YLL002W* (green) and *YLL006W* (purple) were integrated into the neochromosomes respectively or together. Red circle, the spores containing *chrXIILΔ* and the neochromosome.

Next, a chromosome truncation method, which is similar to previous technique for the replacement of telomere^31^, was adopted to truncate the chromosome arm piece by piece (Fig. 2a). In this method, a fragment containing a universal telomere, a marker gene and a homologous region is transformed into yeast to create a new telomere at the left end of *chrXII* by homologous recombination. Depending on the location of the homologous region (HR I–IV, Supplementary Fig. 5a), the left-most arm up to this region will be deleted. We systematically removed the left arm of *chrXII* in quarterly increments to a maximum of the entire arm in a heterozygous diploid strain (Region I-IV, Fig. 2b). Due to deletion of essential genes, the diploid strains with an empty neochromosome backbone (Neo0) produced only two viable spores upon sporulation (Fig. 2b). In contrast, the presence of ptWT10 rescued the lethality of the two spores in three strains, except for the one in which the entire *chrXIIL* was deleted (*chrXIIL*Δ, Fig. 2b), illustrating that the 10 essential genes are insufficient to substitute for *chrXIIL* for viability. It is consistent with the theory that the essential gene set is not sufficient to construct a viable organism with a minimal genome due to the phenotypic consequences of complex genetic interactions on fitness^32^.

Then, we systematically examined the viability of strains containing one more non-essential gene plus the ten essential genes. Among the 64 non-essential genes on *chrXIIL*, we excluded 9 dubious genes, 2 pseudogenes, 3 genes at the telomere and 1 tRNA gene, leaving 49 protein-coding genes to be tested. However, none of the 11-gene combinations were able to produce four viable spores from one tetrad (Supplementary Fig. 5c), suggesting that more genes are required for viability.

Thus, we tried to add two genes. Genetic interactions are quantified by measuring phenotypes of single and double mutants and calculating an interaction factor that reflects any deviation from the expected combined effect of the two single mutants^32^. To date, the investigations of genetic interaction have been extensively conducted in budding yeast, using genome-wide yeast mutant collections and automated colony size-scoring methodology^32^. It has been reported that essential genes participate in more genetic interactions than non-essential genes^32^, raising the hypothesis that the higher importance of the gene, the more genetic interactions may exist. Therefore, we used the number of genes that showed genetic interaction with a target gene (GGI) as a quantitative indicator of genetic interactions. Essential genes did show significantly higher GGI than non-essential genes (Supplementary Fig. 6a). There are 214 non-essential genes (~3.6% in genome) with GGI higher than 489.4 (2-fold to the average GGI of essential genes). Nearly all these genes (98.5%) showed synthetic lethality with other genes, and more than 80% showed decreased vegetative growth or absent respiratory growth when deleted (Supplementary Fig. 6b, c), providing additional evidence for the importance of these genes. Among the nonessential genes on *chrXIIL*, *YLL002W* and *YLL006W* met this threshold (Supplementary Table 1). *YLL002W* was deleted in the strain Δ1 which showed severe growth defects, and it also showed synthetic lethality with 29 other genes (Supplementary Fig. 5, Supplementary Table 1). *YLL006W* was synthetic lethal with 9 other genes, including another non-essential gene *YLL040C* on *chrXIIL* (Supplementary Table 1). So, we chose *YLL002W* and *YLL006W* for further testing.

As expected, single mutants of the two genes exhibited impaired cell fitness and the double mutant led to a severe growth defect (Fig. 2c). Therefore, we constructed three versions of neochromosomes containing the 10 essential genes plus either *YLL002W*, *YLL006W*, or both. Excitingly, we found the strain containing ptWT10 plus both *YLL002W* and *YLL006W* generated four viable spores (Fig. 2d), suggesting that a neochromosome containing just the 12 genes is sufficient to substitute *chrXIIL* for yeast viability. We named the neochromosome with 12 genes as ptWT12 (Supplementary Data 1) and the yeast strain carrying ptWT12and *chrXIIL*Δ as yWT12.

## Additional genes are needed to restore robust cell fitness

**Fig. 3:**
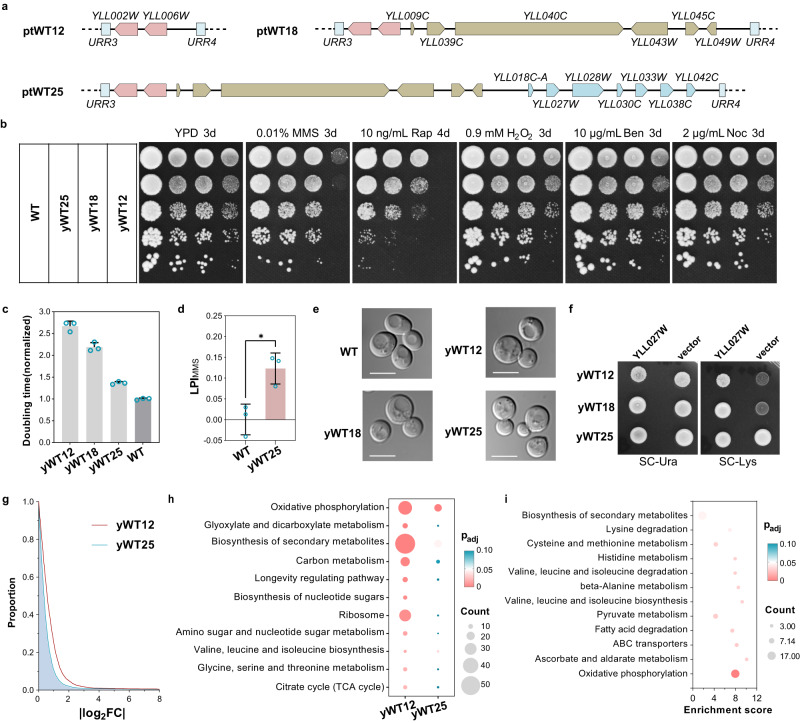
Restoration of cell fitness using a simplified gene set. **a** Schematic diagrams of additional genes in the three neochromosomes besides the essential genes. **b** Fitness analysis of strains carrying assembled neochromosomes under various conditions. WT, BY4741. **c** Doubling times of corresponding strains in SC medium (*n* = 3). The average doubling time of BY4742 was set to 1.0. The data are presented as the mean and SD. Source data are provided as a Source Data file. **d** The logarithmic phenotype index (LPI) of BY4742 (*n* = 3) and yWT25 (*n* = 3) under MMS condition. The data are presented as the mean and SD. Unpaired t-test (two-tail) was used compare the two groups, *p* = 0.0155. The LPI_MMS_ significantly greater than zero indicates the resistance phenotype of corresponding strain. Source data are provided as a Source Data file. **e** Cell morphology of indicated strains. WT, BY4742. Scale bars, 5 μm. Images are representative of at least three independent samples. **f** Lysine auxotrophy due to *yll027w deletion*. *YLL027W* is expressed in a centromeric plasmid under its native promoter and terminator. **g** Transcriptome-wide perturbation of yWT12 and yWT25. BY4742 was used as the control for normalization. X axis represents |log_2_FC|. Y axis represents the percentage of genes with |log_2_FC|> the value of X axis. **h, i** KEGG pathway enrichment analysis of the differentially expressed genes in strains, using clusterProfiler package to calculate Benjamini-Hochberg adjusted *p* values. All eleven terms with adjusted *p* value < 0.05 in yWT12 were shown in (**h**). All twelve terms with adjusted *p* value < 0.05 in yWT25 were shown in (**i**). Bubble size indicates the gene count and the color reflects the adjusted *p* value.

Although yWT12 is viable, it grew poorly even in rich medium (Fig. 2d). To understand how the fitness of yWT12 was compromised, we tried to add back additional non-essential genes from *chrXIIL*, prioritizing those with high GGI value. Since 91.2% of non-essential genes with high GGI (244.7 < GGI < 489.4) also showed synthetic lethality with other genes (Supplementary Fig. 6b), the five genes, *YLL049W, YLL039C, YLL043W, YLL040C, and YLL045*, were chosen. In addition, among the genes in region IV, deletion of *YLL009C* also led to obvious growth defects (absent respiratory growth, Supplementary Table 1), and therefore, it was also included. The six genes, including their regulatory sequences, were amplified from the wild type genome, and incorporated into the neochromosome to construct ptWT18 (Fig. 3a) and the corresponding strain yWT18 (ptWT18, *chrXIIL*Δ). The successful construction of the neochromosome and strains were verified by sequencing (Supplementary Data 1).

As shown in Fig.3b, introducing the six genes could obviously improve the growth of cells, not only on rich medium, but also under the chemical stresses (DNA damaging agent MMS and mTOR inhibitor rapamycin). All of the strains showed no obvious sensitivity to the oxidative stress (H_2_O_2_) or the anti-microtubule drugs (benomyl and nocodazole). Next, we measured the growth rates of these strains in synthetic complete (SC) medium. The doubling time of yWT12 was lengthened to over twice that of the wild type. It was much reduced for yWT18 but remained longer than BY4742 (Fig. 3c).

Besides the genes in ptWT18, there are eight growth-related genes on *chrXIIL* (Supplementary Table 1). Seven out of the eight genes were also retained in ZLY348, the strain with a compacted *chrXIIL* (~40% removal of *synXIIL* sequences) but retaining wild type-like growth on YPD at 30 °C^11^. Therefore, we added them to construct ptWT25 and the strain yWT25 (ptWT25, *chrXIIL*Δ, Fig. 3a, b, Supplementary Data 1), and the doubling time of yWT25 was much shorter than yWT18 (Fig. 3c). yWT25 cells also showed higher resistance to rapamycin than yWT18, and even better resistance to MMS than BY4742 (Fig. 3b, d and Supplementary Fig. 6d, e).

In addition, we analyzed whether there were morphological changes among these strains. As shown in Fig. 3e, the cells of yWT12, yWT18 and yWT25 all looked similar, rounder than BY4742. Interestingly, neither yWT12 nor yWT18 could grow on the synthetic medium without lysine while yWT25 did (Fig. 3f). This phenotype may result from the removal and subsequent re-incorporation of *YLL027W*, the null mutant of which leads to lysine auxotrophy^33,34^. Consistently, expression of *YLL027W* in yWT12 or yWT18 did recover growth on the selective medium (Fig. 3f). In accordance with above phenotypes, the transcriptomic perturbations observed in yWT12 were evidently restored in yWT25 (Fig. 3g). And the majority of genes within the significantly enriched pathways of yWT12 did not exhibit differential expression in yWT25 (Fig. 3h, Supplementary Data 2).

We also tried to further remedy the fitness of yWT25 by adding more genes. From the genes remained in ZLY348, we selected the top five genes exhibiting GGI > 129.6 to construct ptWT31 and the strain yWT31 (ptWT31, *chrXIIL*Δ, Supplementary Fig. 6f, Supplementary Data 1). *YLL013C* (*PUF3*), a gene that encodes an mRNA binding protein involved in mRNA decay processes and known to have over 2000 putative mRNA targets^35^, was also included (Supplementary Fig. 6f). However, no significant improvements of cell growth in YPD were observed in yWT31(Supplementary Fig. 6g). Subsequently, we looked into the transcriptomic data and found that the differentially expressed genes in yWT25 are highly enriched in oxidative phosphorylation (Fig. 3i), including the removed *YLL041C* (*SDH2*). And 25.6% (21/82) of the overlapping differentially expressed genes in yWT12 and yWT25 were implicated in the interaction network with *YLL041C* (*SDH2*) and *YLL013C* (*PUF3*) (Supplementary Fig. 6h, i). Therefore, we incorporated the two genes to build ptWT27 and the strain yWT27 (ptWT27, *chrXIILΔ*, Supplementary Fig. 6j, Supplementary Data 1). Strikingly, yWT27 exhibited a significant recovery of growth in YPD (Supplementary Fig. 6j). This result highlights the potential of transcriptomic data for debugging fitness defects.

Together, these results suggest that although only 12 genes are required for cell survival, additional genes are needed to maintain relatively robust growth. The principles used in this study are useful to identify critical genes to improve strain fitness.

## Altered metabolic profiles contribute to the growth differences between yWT12 and yWT25

**Fig. 4:**
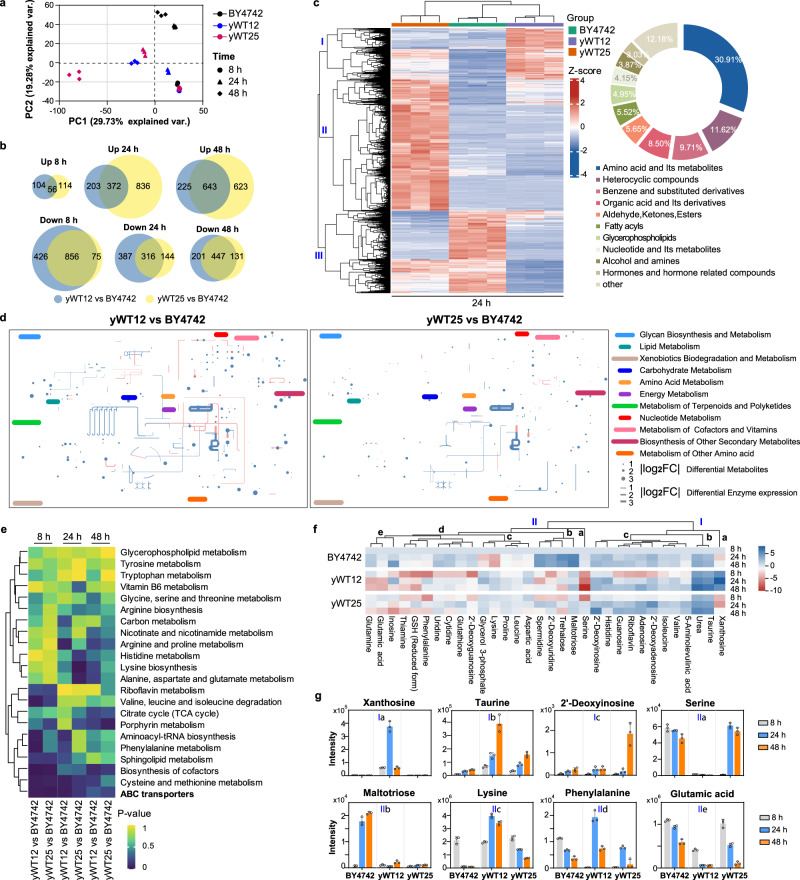
Untargeted metabolomics reveals altered metabolic profiles among yWT12, yWT25 and BY4742. **a** Principal component analysis of metabolite composition using untargeted metabolomics in BY4742, yWT12 and yWT25 collected at 8 h, 24 h, and 48 h. **b** Differentially increased or decreased metabolites at different growth phases shown in Venn diagrams (|log_2_FC| > 1, and one-way ANOVA *t*-test *P* < 0.05). Three biological replicates were conducted. **c** Heatmap of differential metabolites (left) and the proportion of different types of differential metabolites (right) in BY4742, yWT12, and yWT25 at 24 h. The color code in heatmap denotes Z-scaled values of metabolites after correction of confounders. **d** Differential metabolites (dots, 8 h) and differentially expressed metabolic genes (lines) of yWT12 and yWT25 mapped to the yeast metabolic network using iPath3.0. Red and blue dots/lines represent significantly upregulated or downregulated metabolites/genes. Radius/thickness of dots/lines represents |log_2_FC| of metabolites/genes. **e** KEGG pathways that were significantly altered in different strains are indicated. Significantly enriched pathways are identified with a hypergeometric test’s *p*-value for given metabolites. **f** The abundance of DMs belonging to the ABC transporters-dependent pathway was assessed. The abundance of each metabolite was compared to that in BY4742 at 8 h. The color scale indicates the log_2_FC, with blue indicating increased abundance and red indicating decreased abundance. **g** Representative DMs enriched in the ABC transporters-dependent pathway that foster or limit the growth of different strains (*n* = 3). The data are presented as the mean and SD. Source data are provided as a Source Data file.

The enrichments of differentially expressed genes in yWT12 and yWT25 across various metabolic pathways signify the profound metabolic alterations. We employed untargeted metabolomics to investigate the metabolic profiles of the BY4742, yWT12, and yWT25 strains with different growth time in SC medium (8 h, 24 h and 48 h, Supplementary Fig. 7a). Principal component analysis (PCA) has been widely used as a multivariate method in metabolomics analysis, which is a key tool to identify patterns and outliers in the metabolomics datasets^36^. PCA of the metabolites composition among the three strains showed that the first principal component (PC1) versus PC2 accounted for over 49% of the total variation (Fig. 4a), which revealed a shift in metabolite profiles over time. Reliable separations among the three strains were observed at 24 h and 48 h (Fig. 4a), with an increased number of differential metabolites (DMs) found in the yWT12 and yWT25 along with culture time as compared with BY4742 (Fig. 4b). The number of elevated DMs in yWT12 increased progressively over time, with 160, 575, and 868 increased metabolites at 8 h, 24 h, and 48 h, respectively, while the number of decreased DMs declined gradually (Fig. 4b). Similar findings were observed in yWT25, except that more DMs were decreased at 48 h (Fig. 4b).

Unsupervised hierarchical clustering of the metabolome revealed distinct clusters of metabolites at each time point, with BY4742 exhibiting more accumulated metabolites at 8 h, and yWT25 displaying more accumulated metabolites at 24 h and 48 h (Fig. 4c and Supplementary Fig. 7b, c). At 24 h, DMs in cluster II was over-accumulated in yWT25, while DMs in cluster I and III exhibited high accumulation in yWT12 and BY4742 (Fig. 4c). The DMs were classified into ten superclasses, with amino acid and its metabolites being the most prevalent DMs and accounting for over 30% of the total DMs at 24 h (Fig. 4c). However, the metabolites within each cluster were involved in all ten superclasses, revealing a broad range of metabolic fluctuations during the growth of these strains. Visualization of the differentially expressed enzymes and DMs (8 h) by mapping them to the metabolic network using iPath3^37^ revealed that the metabolic alterations observed in yWT12 were markedly restored in yWT25 (Fig. 4d). Notably, while differentially expressed enzymes in yWT25 highlighted changes in energy metabolism and amino acid metabolism, DMs in yWT25 were distributed throughout the metabolic network, similar to those in yWT12 (Fig. 4d).

Further analysis of the DMs using KEGG pathway enrichment revealed that various metabolic pathways were affected during the growth of the different strains (Fig. 4e). ABC transporters, which are involved in the transport of diverse substrates across membranes, were found to be significantly affected (Fig. 4e). 32 DMs were enriched in ABC transporters-dependent pathway (Fig. 4f). Metabolites in cluster I of this pathway, such as xanthoside and taurine (Ia and Ib), and 2’-deoxyinosine (Ic), were particularly abundant in yWT12 and yWT25, respectively (Fig. 4f, g, and Supplementary Fig. 7d), indicating a potential defects in their utilization or transport outside of the cell, which could impede their growth. In contrast, metabolites in clusters IIa and IIb were scarce in yWT12 or yWT25 (Fig. 4f, g, and Supplementary Fig. 7d). For instance, serine was barely detectable in pWT12, while maltotriose was found to be only minimally accumulated in both pWT12 and pWT25 (Fig. 4g), which indicated a potential disability in biosynthesis or transport inside of the cell in these strains. Metabolites in clusters IIc-e showed different abundance levels in different strains (Fig. 4f, g, and Supplementary Fig. 7d), suggesting differences in uptake or metabolic capacity, which could influence growth rates. These findings highlight the importance of ABC transporters for the growth of the modified strains. Consistently, differentially expressed genes in yWT25 were also enriched in the ABC transporters pathway (Fig. 3i). Together, introducing genes involved in ABC transport could be a potential way to further improve cell growth.

## Reconstruct transcription units using exogenous artificial sequences

Given that the native genes could be relocated to the neochromosome without disruption of core function, we next asked if both the coding and regulatory sequences are able to be reconstructed with completely synthetic ones. For the 25 genes in ptWT25, we systematically reconstructed them using the following principles: For coding sequences (CDS), the optimized DNA sequences were generated using GeneDesign software as before^20,38^, which employs a radical codon compression scheme, in which only one optimal codon is used for each amino acid (Supplementary Fig. 8a). And the optimal codon for each amino acid was defined by the highest relative synonymous codon usage value in highly expressed genes in yeast genome^20,38^. Based on previous studies^12–15^, 44 short synthetic promoters and 28 short synthetic terminators with glaring variances in sequence and a variety of expression activities were selected for the promoter and terminator (Supplementary Data 3). Each part was synthesized and cloned into the YeastFab vectors we developed previously^25^.

**Fig. 5:**
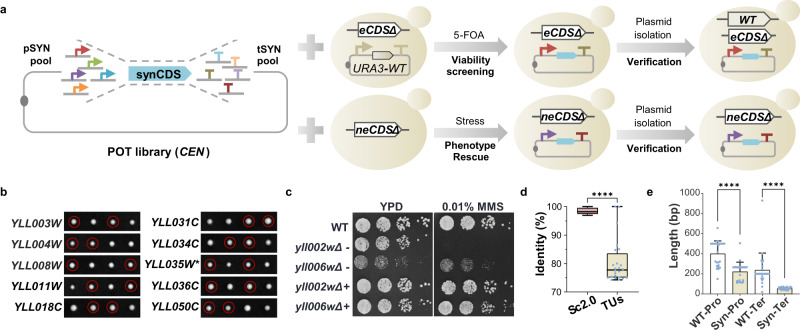
Reconstruction of transcription units. **a** Strategy to identify functional synthetic promoters and terminators to support both essential and nonessential genes. eCDS, CDS of essential gene. neCDS, CDS of nonessential gene. *URA3*-WT, the *CEN* plasmid containing wild-type essential gene and *URA3*. **b** Functional analysis of reconstructed genes by tetrad analysis. The corresponding heterozygous diploid containing a reconstructed TU (rTU) was sporulated and dissected. One representative tetrad was shown for each strain. For *YLL035W*, the ATG codon was mutated (marked with *) to avoid impact on the expression of *YLL036W*. Red circle, the spores containing disrupted native gene and reconstructed TU. **c** Functional complementation test of reconstructed *YLL002W* and *YLL006W*. WT, BY4741. + and − indicate strains with or without the reconstructed TU. **d** Comparison of sequence identity in CDS between the 24 rTUs and corresponding genes in Sc2.0 project. The bounds of the box were the upper and lower quartile with the median value in the center. The whiskers indicated the minimum and maximum. **e** Length comparison of Pro and Ter between the 24 rTUs and the native ones used in ptWT25. The data are presented as the mean and SD. Unpaired *t* test (two-tail) was used for comparison in (**d**) (*p* = 3.57E-11) and (**e**) (*p* = 2.15E-06 for Pro, *p* = 2.77E-05 for Ter). Source data are provided as a Source Data file.

To obtain functional combinations of promoter, CDS and terminator for each gene, we mixed the promoter-, terminator-containing plasmids together with a particular CDS-containing plasmid to assemble a pool of TUs, which were subsequently transformed into the corresponding haploid deletion mutant (Fig. 5a). For essential genes, plasmid shuffling was performed to identify the viable clones. For non-essential genes, fitness change under a particular growth condition was identified for each knockout strain and clones that were able to restore growth to that of wild type were collected. The plasmids were extracted, transformed into *E. coli* and subsequently sequenced to obtain the identity of promoter and terminator. Combinations of promoter and terminator for each gene were re-tested to confirm their functionality, either by tetrad-based analysis of reconstructed essential genes for the ability to support cell survival (Fig. 5b) or by serial-dilution analysis (Fig. 5c, Supplementary Fig. 8 and Supplementary Data 3). Using promoters and terminators as different as possible for each gene, we successfully reconstituted 21 TUs except *YLL031C*, *YLL028W*, *YLL003W* and *YLL039C*. For both *YLL031C* and *YLL028W*, we found the recoded CDS failed to complement the function of native CDS, either losing cell viability or failing to grow under stress conditions. In addition, we discovered that *YLL003W*, a gene required for G2/M transition, lost function when constitutively expressed. To obtain a functional TU, the promoter of *SWI6*, a transcription factor activating transcription during G1/S transition, was employed which, luckily, could restore the function of *YLL003W*^20^. As for *YLL039C (UBI4)*, we failed to assemble the recoded gene, potentially due to its repetitive nature since it encodes five head-to-tail ubiquitin repeats within CDS^39^.

We compared sequence changes in the 24 TUs after reconstruction to those in wild type or Sc2.0 strain. At first, significant reduction of sequence identities in CDS was present. The Sc2.0 sequences share over 95% identity to wild type, whereas the mean identity of the reconstructed genes dropped below 80% (Fig. 5d). As for the regulatory sequences, not only totally different DNA sequences were used (Supplementary Data 3), the lengths of the synthetic promoters and terminators were also much shorter than the counterparts in ptWT25 (Fig. 5e).

## Replacement of *chrXIIL* by a completely revamped neochromosome

Given that each refactored gene is able to function similarly to the native one, we tested whether combinations of these genes could replace *chrXIIL* entirely. The ptSYN10 neochromosome with ten refactored essential genes was constructed (Supplementary Fig. 9a) and those essential genes were deleted from the native chromosomal locations to get the strain ptSYN10-10KO (Supplementary Fig. 9b). As shown in Supplementary Fig. 9b, ptSYN10 was able to support the viability of the ptSYN10-10KO strain, but the cells showed growth defects on either YPD or upon treatment of different drugs. RNA-seq analysis indicated that, in contrast to the neochromosomes made up of essential genes with native regulatory sequences (Supplementary Fig. 4a), nearly all the intergenic sequences of the synthetic genes on ptSYN10 were highly transcribed (Supplementary Fig. 9c). To further look into the transcriptional fidelity of each gene, ptSYN10 in BY4742 was also analyzed by Iso-seq (Supplementary Fig. 9d). Intrusive readthrough was identified into adjacent transcription units (Supplementary Fig. 9d). Moreover, many anti-sense transcripts appeared, especially for *syn035w* and *syn034c* (Supplementary Fig. 9d). These results suggested that the short synthetic terminators might not be effective at terminating transcription, and insertion of artificial DNA might bring in sequences with unexpected promoter activities.

**Fig. 6:**
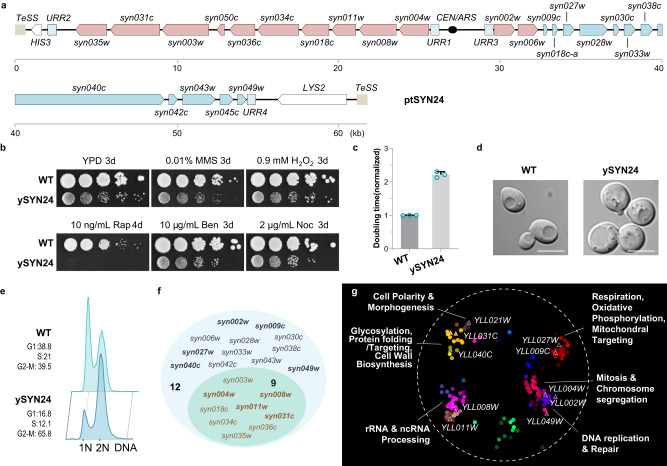
Functional replacement of *chrXIIL* with a completely revamped neochromosome. **a** Schematic representation of gene arrangement on ptSYN24 neochromosome. **b** Fitness of ySYN24 in different growth conditions. WT, BY4741. **c** The doubling time of ySYN24 (*n* = 3) in YPD. WT, BY4742 (*n* = 3). Source data are provided as a Source Data file. The data are presented as the mean and SD. **d** The cell morphology of ySYN24. WT, BY4742. Scale bars, 5 μm. Images are representative of at least three independent samples. **e** FACS analysis after propidine iodide staining on asynchronous cells. WT, BY4742. **f** Differentially expressed genes on ptSYN24 in ySYN24. The nine essential TUs, whose counterpart in ptWT10U-10KO were also differentially expressed, were labeled in brown. **g** Functional enrichment analysis of the differentially expressed genes in ySYN24. The differential genes were mapped to a global similarity network annotated using SAFE. The colors represent different function domains. Triangles with white rims indicate genes located in *chrXIIL*.

We next built a neochromosome (ptSYN12) carrying the same 12 genes as ptWT12 but using recoded sequences (Supplementary Fig. 9e). Unfortunately, unlike its wild type counterpart, the ptSYN12 neochromosome was unable to substitute *chrXIIL* for survival, although it did support several cell divisions after spore germination (Supplementary Fig. 9f). Next, based on what we learned above using native genes, we constructed the ptSYN24 neochromosome (Fig. 6a), which contains 24 recoded TUs from ptWT25 without *UBI4*. Excitingly, we found ptSYN24 could support viability in spores with *chrXIIL*Δ (Supplementary Fig. 9g). These strains were designated as ySYN24 (Supplementary Data 1).

Subsequently, several characterizations on ySYN24 were carried out. At first, it showed growth defects on YPD plates and severe sensitivity to rapamycin, while less dramatic fitness defects were detected under other conditions (Fig. 6b). Consistently, its doubling time was more than twofold longer than that of wild type (Fig. 6c). In addition to the round shape like yWT25, the cell size was slightly also enlarged in ySYN24 (Fig. 6d and Supplementary Fig. 9h). The fraction of cells with 2 N DNA in log-phase population was significantly higher than that of BY4742 (Fig. 6e), suggesting a cell-cycle defect. In addition, ySYN24 could not be arrested as efficiently as wild type under nutrient depletion conditions (Supplementary Fig. 9i).

Furthermore, we determined the transcriptional profile of ySYN24 (Fig. 6f, g) and found that over three-quarters of genes on the neochromosome (21/24), including nine essential genes, were overexpressed (Fig. 6f, Supplementary Data 2). To visualize the functional enrichments of the differentially expressed genes in ySYN24, we mapped them to a yeast global genetic interaction similarity network annotated by spatial analysis of functional enrichment (SAFE)^40^ and labeled the genes on *chrXIIL* (Fig. 6g). These genes were enriched in several bioprocesses, such as glycosylation, cell wall biosynthesis, rRNA processing, DNA replication and repair, mitosis, morphogenesis, and respiration. Four essential genes (*YLL031C*, *YLL008W*, *YLL011W,* and *YLL004W*) and six non-essential genes (*YLL040C*, *YLL027W*, *YLL009C*, *YLL002W, YLL049W,* and *YLL021W*) were clustered in corresponding bioprocesses. Misexpression or deletion of these genes may collectively contribute to the defects of ySYN24, such as round cell shape caused by the removal of *YLL021W*^41^. Fine-tuning the expression of the TUs for the other nine genes could be a promising approach to optimize the fitness of ySYN24.

## Discussion

The synthetic yeast genome has provided us a foundation to probe the function of the eukaryotic genome with various design principles^8^. In this study, using *chrXIIL* as an example, we explored the possibility of simplifying the yeast genome by targeted knockout and bottom-up gene replenishment, extended from previous results of random deletions using SCRaMbLE^11^. We successfully removed about four-fifths of the sequences in the chromosome arm and generated a strain that could survive with only 12 genes (10 essential genes plus *YLL002W* and *YLL006W*). In addition, we demonstrated that, for most genes tested in this study (21/24), they could be reconstructed using synonymously recoded ORFs and synthetic regulatory DNA to retain their basic function. In particular, we found that aggressive reprogramming of the coding sequences, that is, to encode each amino acid with the same codon, can be tolerated, at least on this small scale by the yeast. However, the fact that two genes with recoded CDSs and one gene with synthetic promoters resulted in lethality or some phenotypic changes in the mutants, suggests that caution must be taken when redesigning synthetic genes with artificial sequences.

The successful synthesis of viral genomes, bacterial genomes and yeast chromosomes imply that chemically synthesized genomes can support life as well as wild-type genomes^42–44^. Here, we demonstrate that the functions of wild-type chromosomes can be assigned to neochromosomes with simplified gene contents, arrangements and sequences. By utilizing the GGI and growth phenotype of nonessential genes, we constructed strains resistant to various stresses, such as yWT25, with only about one-third genes on *chrXIIL*. Through transcriptome data comparison, we improved the fitness of yWT25 using only two additional genes. Combining these principles with multi-omics data can facilitate the design of more simplified neochromosomes with preferred features, which can be used to study the central network of yeast genome and even potentially bypass the need of certain essential genes. In addition, the results in this study and several previous studies including relocating the rDNA locus^29,45^ and minimizing chromosome number in yeast^46–48^ all suggest that the specific organizational form of the yeast genome, including the chromosome number, gene organization and genome 3D structure, might only play minor roles in the basic functions of the yeast genome. This might be the consequence of a small genome and mainly short-range gene regulation in yeast^49^.

In nature, the 20 amino acids are encoded by 61 genetic codons, that is, most amino acids are encoded by more than one codon. Codon usage differs among organisms, and two of the 61 sense codons were removed from a synthesized *E. coli* genome^19^, suggesting the number of codons can be reduced. Theoretically, only 20 codons are needed to encode 20 amino acids. It may, however, be difficult to achieve this minimal codon set because of potentially important roles of codon degeneracy in gene expression regulation^16^. It is surprising that, in this study, most of the synthetic genes using the audacious rule that one amino acid is encoded by only one codon remain functional, suggesting the natural genome very plastic.

To prevent unexpected deleterious effects on gene function and to reserve the wild type-like fitness in the synthetic strains, no sequence engineering was applied to the promoters and terminators in Sc2.0^8^. Synthetic promoters and terminators designed based on the architecture of native ones yield diverse but reproducible expression levels of fluorescent proteins and exogenous enzymes, which helps a lot in deciphering the eukaryotic gene-regulatory logic and metabolic engineering^12–14,50,51^. In this study, we showed that, although no sequence homology exists between these synthetic promoters and terminators and the native ones, they actually can also be used to build functional TUs, including the essential and nonessential ones, demonstrating the high plasticity of regulatory sequences.

In summary, as a pilot of the Sc3.0 project^52^, this study presents the most radical changes we have ever made to a chromosome arm. It indicates substantial plasticity of the yeast genome and suggests the feasibility of using computationally designed sequences to build functional eukaryotic genomes when we acquire enough artificial regulatory elements and knowledge of the complex co-regulation network in future. At the same time, the fitness defects of the strain with totally artificial sequences and the failure to reprogram several elements suggest that current strategies used in this study will be risky to scale up to genome-wide redesign at the moment. Natural sequences from related organisms may provide one of better solutions for the reprogramming of the whole yeast genome.

## Methods

### Strains and growth media

The yeast strains used in this paper were derivatives of BY4741/2 or *synXIIL*^11^. Standard methods for yeast culture and transformation were applied. Targeted knockout methods were applied for deletion through homologous recombination. The strains generated in this study are listed in Supplementary Data 4. For phenotypic analysis of strains, cells were cultured in YPD medium or YPD medium containing 0.9 mM H_2_O_2_, 2 μg/mL nocodazole, 10 ng/mL rapamycin, 0.01% methyl methanesulfonate, or 10 μg/mL benomyl separately. For the function analysis of reconstructed TUs, cells were cultured on synthetic medium with or without additional drugs. Particularly, the carbon source in SCG-Ura was 3% glycerol, other than 2% glucose in other medium.

### Construction of neochromosomes

Construction of neochromosomes include four steps: segment preparation, yeast transformation, PCR identification and nanopore sequencing.

#### Segment preparation

All segments used for assembly contained at least 40 bp sequences overlapped with their adjacent segments at each side. The segments were released from plasmids using *NotI* digestion or PCR amplified using *synXIIL* genome as template. Consistent with previous study^25^, the native promoters were defined as 500 bp or the intergenic regions, whichever is shorter. Similarly, the native terminators defined as 200 bp downstream or the intergenic regions, whichever is shorter.

(A) A total of 11 segments were used for assembling the ptWT10 neochromosome. Segment L and R were released from the constructed plasmids using *NotI* digestion, segment M was PCR amplified using a yeast strain containing a yeast artificial chromosome (Fig. 1A). Segment 1–7 were amplified using *synXIIL* genome as template. The coordinates for these segments in the synXII reference sequence^29^ are: segment 1, encoding *YLL050C*, 27,316-28,330; segment 2, encoding *YLL034C*, *YLL035W* and *YLL036C*, 54,606–61,379; segment 3, encoding *YLL031C*, 64,787–68,394; segment 4, encoding *YLL018C*, 96,751–100,011; segment 5, encoding *YLL011W*, 115,173–117,239; segment 6, encoding *YLL008W*, 119,397–122,283; segment 7, encoding *YLL003W* and *YLL004W*, 128,529–134,272. In addition, a fused linker containing sequences of both URR3 and URR4 was also used to ligate segment M and R.

(B) In total, eight segments were used for assembling the ptWT10U neochromosome. Segment L, R, and M were prepared using the same method as ptWT10. Since the promoters of *YLL035W* and *YLL036C* share common sequence and some important elements of the promoter of *YLL036C* are located in the coding region of *YLL035W*, the promoters of *YLL035W* and *YLL036C* were changed to p*CYC1* and the terminator of *YLL035W* was changed to t*CYC1*^20^. Segments 1–4 were amplified using the gDNA of BY4742 containing ptWT10 as template. The coordinates for these segments in the ptWT10 reference sequence are: segment 1, encoding *YLL050C* and *YLL036C*, 2532–5168; segment 2, encoding *YLL035W*, 5492–7390; segment 3, encoding *YLL034C*, *YLL031C* and *YLL018C*, 7545–17,189; segment 4, encoding *YLL011W*, *YLL008W, YLL004W* and *YLL003W*, 17,190–27,887. The same fused linker in ptWT10 was also used.

(C) The ptWT12 was constructed by integrating a segment containing *YLL002W*, *YLL006W* and *KanMX4* at the right arm of ptWT10. The coordinates for *YLL002W* and *YLL006W* in *chrXII* (NC_001144.5) are: *YLL002W*, 146,041–147,802; *YLL006W*, 136,300–137,939.

(D) The ptWT18/25/27/31 were constructed similarly as ptWT12. Segment 1 containing eight nonessential genes and a *KanMX4* selection marker was integrated between URR3 and URR4 of ptWT10 firstly (ptWT18), then segment 2, containing 7 nonessential genes and a *NatNT2* selection marker, was integrated at the middle of URR4 (ptWT25). The coordinates for these 15 nonessential genes in *chrXII* (NC_001144.5) are: *YLL002W*, 146,041–147,802; *YLL006W*, 136,300–137,939; *YLL009C*, 131,005–131,728; *YLL039W*, 63,593–65,707; *YLL040C*, 54,011–63,925; *YLL043W*, 49,438-52,087; *YLL045C*, 47,659–49,129; *YLL049W*, 40,179–41,381; *YLL018C-A*, 108,476–109,472; *YLL027W*, 86,903–88,356; *YLL028W*, 84,304–86,765; *YLL030C*, 80,156–81,197; *YLL033C*, 72,909–74,302; *YLL038C*, 65,575–67018; *YLL042C*, 51,877–53,090. For ptWT31, four segments containing six non-essential genes were integrated to replace the *NatNT2* selection marker of ptWT25. The coordinates for the 6 nonessential genes in *chrXII* (NC_001144.5) are: *YLL032C*, 74,070–77,247; *YLL029W*, 80,961–83,910; *YLL019C*, 105,486-108,399; *YLL014W* and *YLL013C*, 120,822–125,214; *YLL001W*, 147,390-150,363. For ptWT27, the segment containing 2 nonessential genes and *URA3* was integrated into ptWT25 to replace *KanMX4*. The coordinates for the 2 nonessential genes in *chrXII* (NC_001144.5) are: *YLL013C*, 121,837–125,222; *YLL041C*, 52,931–54,211.

(E) In total, seven segments were used for assembling the ptSYN10 neochromosome. Segments L, R, and M were prepared using the same method as ptWT10 and ptWT10U. Segments 1–3 were released from the synthesized plasmids using *NotI* digestion. The same fused linker in ptWT10 was also used. The coordinates for these segments in the ptSYN10 reference sequence are: segment 1, encoding *syn035w* and *syn031c*, 2536–8907; segment 2, encoding *syn003w*, *syn050c*, *syn036c*, and *syn034c*, 8097–17,510; segment 3, encoding *syn018c*, *syn011w*, *syn008w* and *syn004w*,17,5111–25,503.

(F) 7 segments were used for assembling the ptSYN12 neochromosome. Segment L, R and M were prepared using the same method as ptWT10 and ptWT10U. Segments 1–3 were prepared using the same method as ptSYN10. Segment 4 was the fused product, including URR3, URR4, and the DNA encoding *syn002w* and *syn006w*. The coordinates for these segments in the ptSYN12 reference sequence are: segment 1, encoding *syn035w* and *syn031c*, 2536–8907; segment 2, encoding *syn003w*, syn050c, *syn036c* and *syn034c*, 8097–17,510; segment 3, encoding *syn018c*, *syn011w*, *syn008w*, and *syn004w*, 17,511–25,503; segment 4, encoding *syn006w* and *syn002w*, 29,363-32,365.

(G) There were two steps to construct the ptSYN24 neochromosome based on ptSYN10. The first step integrated a segment containing *KanMX4* selection marker franked with two *I-SceI* sites between URR3 and URR4. Six segments for assembling the ptSYN24 neochromosome were transformed into the first-step strain expressing I-SceI restriction enzyme. Segment 1, 2, and 4 were released from the plasmids containing synthetic genes by *BsaI* digestion. Segment 3 was amplified using the plasmid containing *syn040c*. Segment 5 and 6 were amplified by overlapping PCR. The coordinates for these segments in the ptSYN24 reference sequence are: segment 1, encoding *syn002w, syn006w, syn009c, syn018c,* and *syn027w*, 28,813–34,513; Segment 2, encoding *syn028w, syn030c, syn033w* and *syn038c*, 34,474–39,327; Segment 3, encoding *syn040c*, 39,284–49,041; Segment 4, encoding *syn042c*, *syn043w, syn045c,* and *syn049w*, 48,764–54,652; segment 5, 34,194–34,793; segment 6, 39,003–39,602.

#### Yeast transformation

the protocol described in our previous paper^29^ was used for yeast transformation. Briefly, 5 mL log-phase cells with OD_600_ ~ 0.4–0.6 were harvested and washed with ddH_2_O and 0.1 M LiOAc/TE, respectively. And 100 μL 0.1 M LiOAc/TE were used to resuspend the cells. DNA fragments or plasmids were added to the cells. The mixture, including 312 μL PEG3350, 41 μL 1 M LiOAc and 25 μL ssDNA (which were boiled at 100 °C for 10 min and cooled on the ice for 5 min before used), were added and mixed. After a 30 min incubation at 30 °C, 50 μL DMSO were added and mixed. Then the tube was subjected to the heat-shock for 15 min at 42 °C. Cells were washed once with 5 mM CaCl_2_ and plated onto the appropriate plates. The plates were cultured at 30 °C for the appropriate time. The linear neochromosomes were usually assembled in BY4742. Then these strains were used to mate with MATa strains containing either multi-gene deletion or truncated *chrXIIL* to construct the diploid strains which could sporulate to generate the haploid strains for functional test of the neochromosomes, such as ptWT10-10KO and ySYN24.

#### PCR identification

primers for synthetic sequence identification (PCRtags) and junction verification were designed and listed in Supplementary Data 5. The specificity of PCRtags was verified using the genomic DNA of strains without the corresponding neochromosome as templates. PCR were performed the same as our previous report^29^. Only colonies positive for all junctions and PCRtags were subjected to further sequencing analysis.

#### Nanopore sequencing

Total DNA was extracted using the QIAGEN Genomic-tips 100/G with Genomic DNA buffer Set following the manufacturer’s instruction. DNA quality was assessed by NanoDrop, gel-electrophoresis and Qubit. Libraries were prepared using the Ligation Sequencing Kit (SQK-LSK109) with the barcoding kits Native Barcoding Expansion 1–12 (PCR-free, EXP-NDB104) and Native Barcoding Expansion 13–24 (PCR-free, EXP-NDB104). Sequencing was performed using the MinlON platform with FLO-MIN106D for a 72-h run. Base calling, adapter removal, and low-quality base filtering based on fast5 files were done by ONT software MinKNOW. The FastQ files were filtered by NanoFilt^53^ to remove short (read length < 500) and low-quality (average read quality score <7) reads. The remaining reads were mapped to reference genome by NGMLR^54^ and assembled into contigs using Canu 2.1.1^55^. Alignments between contigs and reference genomes were generated by MUMmer-nucmer 3.23^56^ with default parameters and were used to create collinearity dot plot by Dot (https://github.com/dnanexus/dot). We divided the reads into two parts: the linear neochromosome and native chromosomes. The mean read-depth of a 1 kb-sized bin for all native chromosomes was normalized to 1 and the ratio of the mean depth of the neochromosome to that of all native chromosomes was calculated as the average copy number of the neochromosome per haploid genome. The reads containing full-length neochromosome were extracted and aligned to reference sequence to infer the actual length of telomere TG repeats (TG repeats to the extremity).

### Pulsed-field gel electrophoresis and Southern blotting

About 2 × 10^8^ cells at stationary phase were fixed in the 0.6% low melting point agarose for each plug and genomic DNA was prepared as described before^29^. Plug samples were resolved on a 1% agarose gel in 0.5 X TBE for 16 h at 14 °C on a BioRad CHEF Mapper XA Pulsed Field Electrophoresis System. The voltage was 6 V/cm, at an angle of 120° and switch time from initial 0.5 s to 1.5 s. The gels after PFGE were washed with ddH_2_O twice and transferred onto Hybond-N+ membrane (Amersham). The samples were UV crosslinked and hybridized with DIG-labeled probes. Five DNA probes were amplified from corresponding linear chromosomes for probe labeling and mixed together for hybridization. Probe preparation, hybridization and detection were conducted with DIG-High Prime DNA Labeling and Detection Starter Kit (Roche, 11585614910). The primers for probes amplification are listed in Supplementary Data 5.

### Stability analysis of assembled chromosomes

Two independent colonies of the targeted strain were inoculated into 5 mL SC-His medium and cultured at 30 °C with shaking at 220 rpm for 24 hrs. Then 5 μL of the culture was added into 5 mL fresh SC-His medium and grew for another 24 h. The cells after 10 days’ passages were plated onto SC-His plates to isolate single colonies. Ten colonies of each parent were picked and cultured separately for gDNA preparation and PCRtag analysis to see whether some deletion events have happened during about 125 generations.

Three out of the twenty colonies were further analyzed by whole genome sequencing using the Miseq platform. Because the neochromosomes are non-essential for BY4742, SC-His medium was used to select the neochromosome. The indicated strain was inoculated into 5 mL SC-His liquid medium and incubated at 30 °C for 12 h with shaking at 220 rpm. 5 × 10^7^ yeast cells were harvested for total DNA extraction using the Monarch® Genomic DNA Purification Kit based on the manufacturer’s instructions. 300-1000 bp libraries were prepared using the Nextera DNA Flex Library Prep Kit for purified genomic DNA and then sequenced on the Illumina Miseq platform with PE250 strategy.

After adapter removal, the low-quality reads were trimmed by Trimmomatic^57^ with parameters “SLIDINGWINDOW:5:20 LEADING:5 TRAILING:5 MINLEN:50”, and the remaining reads were mapped to reference genome with BWA-mem^58^. After marking PCR repeat using GATK-MarkDuplicates, the SNPs were called by GATK- HaplotypeCaller and GATK- GenotypeGVCFs. Structural variations were called using DELLY^59^.

### Serial dilution

The serial dilution was performed as previously mentioned^29^. In short, a single colony was inoculated into 3 mL YPD medium and incubated at 30 °C with shaking at 220 rpm for 24 h. The OD_600_ was measured, and the overnight cultures were diluted by sterile water to OD_600_ = 0.2. After four tenfold gradient dilutions, well-mixed cells were dropped onto indicated plates. The plates were cultured at 30 °C for appropriate time unless specifically mentioned.

### Doubling time assay

The measurements of growth curve were performed as before^60^. Briefly, the log-phase cells were diluted with fresh medium to the same density, and 100 µL of the diluted cells were added to each well of the Costar clear polystyrene 96-well plates. For each culture, three or four technical replicates were examined. The 96-well plate sealed with Breathe-Easy membrane (Sigma, MKBZ0331) was cultivated in an Epoch2 microplate photometer (BioTek) at 30 °C for 36–48 h in a selected medium. The OD_600_ of each well was recorded every 10 min. The doubling time (*D*) of each strain were acquired using the GraphPad Prism 8 software.

To analyze the resistance phenotype of yWT25, strain fitness was measured using logarithmic strain coefficients (LSC) and logarithmic phenotype index (LPI)^61,62^. The growth of cells from three colonies of BY4742 or yWT25 in YPD with or without MMS were analyzed. Doubling times were used to generate LSC and LPI based on the following formula:

$${{{{{{\rm{LSC}}}}}}}_{i-j}={{{{\mathrm{ln}}}}}\left(\frac{{{{{{{\rm{D}}}}}}}_{{WT}-j}}{{{{{{{\rm{D}}}}}}}_{i-j}}\right)$$

and

$${{{{{{\rm{LPI}}}}}}}_{i-j}={{{{{{\rm{LSC}}}}}}}_{i-j}-{{{{{{\rm{LSC}}}}}}}_{i-{YPD}}$$

where D_*WT-j*_ represents the average doubling time of the three BY4742 strains under condition *j*, D_*i-j*_ represents the doubling time of strain *i* under condition *j*. The LPI_MMS_ significantly greater than zero indicates the resistance phenotype of corresponding strain.

### Differential fitness scoring

To derive quantitative estimates of strain fitness, the same batch of original images of agar plates after 3-day culture at 30 °C captured by the same camera in serial dilution assay were used for scoring. These original images were in the format of.JPG with a resolution of 3168 pixel × 4752 pixel. To meet the resolution restriction of CellProfiler^63^, the original images were unified compressed by 50% before the modified pipeline from the example on the website (https://cellprofiler.org/examples). To identify colonies effectively, we set the “Typical diameter of objects” parameter in the “Identify Primary Objects” step as 2 to 150^64^, and manually checked the positions of the forced spots and the natural spots to avoid misidentification. The “Mean Intensity” value of the spots in the fourth gradient, where the wild-type strain did not reach the up limit of the “Mean Intensity” value, was used as a measure of cell growth for corresponding strains^63,65^. To estimate strain fitness in each condition, the average “Mean Intensity” for the four replicates of wild type cells at different positions in the same plate in each condition were normalized to be 1, and the normalized “Mean Intensity” for strain *i* in condition *j* was defined as the fitness score in condition *j* (*f*_*ij*_). The average fitness of the three replicates for strain *i* on YPD plates was defined as the reference fitness (*f*_*ir*_). Similar to previous study^66^, we calculated the differential fitness score for each strain by measuring the difference between the *f*_*ir*_ and the *f*_*ij*_. The 2way ANOVA analysis was used to calculate the difference significance. The stress conditions included MMS, benomyl, H_2_O_2_ and nocodazole.

### Transcriptome analysis

The non-stranded RNA sequencing libraries were prepared and sequenced by Beijing Novogene Bioinformatics Technology Co., Ltd. using Next® Ultra^TM^ RNA Library Prep Kit for Illumina®. Because of the deletion of a few genes in our strains, we first generated the diminished reference genomes according to our design. We used Cutadapter software^67^ to remove adapters in raw data. HISAT2^68^ and Picard were then used to accomplish the alignments of cleaned reads and remove PCR duplicates. Hereafter, Htseq-count software was employed to calculate read counts of each gene while intersection-nonempty option was set. The downstream statistical analysis was achieved by the DEseq2^69^ package in R. Considering that the remaining fragment of *his3Δ1* in *chrXV* can still be transcribed, we only use the deleted part in *his3Δ1* to quantify *HIS3*. Differential expression in this paper was defined as |log_2_ FC| > 1 and −log_10_ (Adjusted *p* value) > 4.

The transcriptome-wide disturbance was inferred according to |log_2_FC|. For SAFE analysis, the SAFE add-on with default parameters in cytoscape was used^70^. The original network subjected to SAFE was derived from the global genetic interaction similarity network published^40^. The GO biological process annotation was also provided by the SAFE add-on. The transparency of the differentially expressed genes in ySYN24 is set to 255 while the other genes are set to 0.

### Metabolome analysis

Single colonies were inoculated into 5 mL of SC medium for overnight at 30 °C. Each culture was diluted to OD_600_ = 0.1 in fresh SC medium. Cells were collected from 25 mL culture at different time points (8 h, 24 h and 48 h) and extracted for untargeted metabolomics according to the previous methods with modifications^71^. A 1 mL solution (methanol: water = 4:1, V/V) was added into the sample, which were further placed in liquid nitrogen for 5 min and on the dry ice for 5 min, and then were thawed on ice and vortexed for 2 min. This above freeze-thaw circle was repeated three times. The sample was centrifuged at 13.400 g for 10 min and the supernatant was transferred and placed in −20 °C for further analysis. Ultraperformance LC/QTOF MS (UPLC-QTOF-MS/MS) acquisition was applied for untargeted metabolomic analysis. Chromatographic separation of the metabolome was performed on an ACQUITY UPLC BEH C18 (1.8 µm, 2.1 mm * 100 mm; Waters, Milford, MA, USA) with column temperature maintained at 40 °C. Binary mobile phases with phase A of water containing 0.1% formic acid (v/v) and phase B of acetonitrile (0.1 % formic acid) were used for elution, respectively. The column was eluted with the following linear gradient program: 0 min, 5% B; 11 min, 90% B; 12 min, 90%B; 12.1 min, 5% B; and 15 min, 5% B. The flow rate was set at 0.4 ml/min, and the injection volume was 2 μL. The MS data acquisition was operated using the information-dependent acquisition mode using Analyst TF 1.7.1 Software (Sciex, Concord, ON, Canada).

Raw metabolome data was converted into mzML format by ProteoWizard software^72^. Peak extraction, peak alignment and retention time correction were respectively performed by XCMS program. The “SVR” method was used to correct the peak area. The peaks with detetion rate lower than 50% in each group of samples were discarded. After that, metabolic identification information was obtained by searching the laboratory’s self-built database, integrated public database, AI database and metDNA. Data were loaded in R (www.r-project.org) and unsupervised PCA was performed by statistics function prcomp. The hierarchical cluster analysis (HCA) results of samples and metabolites were presented as heatmaps with dendrograms, while pearson correlation coefficients (PCC) between samples were caculated by the cor function in R and presented as only heatmaps. Both HCA and PCC were carried out by R package ComplexHeatmap. Differential metabolites were determined by |log_2_FC| > 1 and *P* value (*P* value < 0.05, Student’s *t* test for two-group analysis and ANOVA for multi-group analysis).

Identified metabolites were annotated using KEGG Compound database (http://www.kegg.jp/kegg/compound/) and annotated metabolites were then mapped to KEGG Pathway database (http://www.kegg.jp/kegg/pathway.html). Significantly enriched pathways are identified with a hypergeometric test’s *P* value for a given list of metabolites^73^. For visualization of DMs in the metabolic network, the KEGG compound IDs of DMs (8 h) are mapped to iPATH3^37^ yeast metabolism pathway map (https://pathways.embl.de/, species filter set to “sce”) to generate dots. The uniprot ID of differentially expressed genes from transcriptomic data are also mapped to the identical map to generate lines.

### Full-length transcriptome analysis

The transcriptomes of indicated strains were sequenced using the PacBio platform at Grandomics Company. Circular Consensus Sequencing (CCS) reads were generated using SMRT-Link (version 8.0.0.80529), with the following modified parameters: “--min-passes 0 --min-length 50 --max-length 21000 --min-rq 0.75”. We used Lima (version 1.10.0, commit SL-release-8.0.0) for Single Cell Full-Length Non-Concatemer (FLNC) reads detection. Lima is integrated into the PacBio official SMRT-Link (version 8.0.0.80529) software package. Lima map 5′ and 3′ primers to CCS reads first, then parse standard pair of 5′ and 3′ primers CCS as the full-length isoform, next trim the primer sequence and polyA tail in each full-length isoform. Here, each isoform was oriented and correspond to cDNA orientation from 5′ to 3′ end.

After FLNC detection, primer and polyA tail trimming, the remaining fraction of each isoform FLNC read was split into wild-type genome derived reads and neochromosome derived reads according to PCRtags using LAST software (https://gitlab.com/mcfrith/last), except the ptSYN10 libraries (alignment methods can distinguish the completely recoded genes on ptSYN10 from the wild-type ones). The neochromosome derived reads or all reads from ptSYN10 libraries were aligned to the designate reference genome with minimap2^74^ (version 2.17-r974-dirty) in spliced alignment mode with parameters: “-ax splice -uf --secondary=no -C5”. Reads with more than 95% identity and 30% coverage of a certain CDS are directly counted from paf files.

To ensure the generation of transcripts with high accuracy, we use cDNA_Cupcake (https://github.com/Magdoll/cDNA_Cupcake) python script “collapse_isoforms_by_sam.py” to collapse redundant isoforms. The “--flnc-coverage” for minimum collapsed reads is set to 5 and the other parameters are set to default. After redundant isoforms collapsing, unique isoforms can be reported as GFF file. A homemade R script is used to illustrate the TSS, TES and transcription direction based on gggenes (https://github.com/wilkox/gggenes).

### Truncation of *chrXIIL*

The four homologous fragments were amplified from the *synXIIL* and respectively cloned into the plasmid with TeSS sequences using the restriction sites, *XmaI* and *SalI*. The TeSS-Marker-HR fragments were released by *BsaI* digestion and transformed into the heterozygous diploid cells (*chrXII* X *synXIIL*). The chromosome arm capped strains were screened with the PCRtags which have been reported to distinguish *chrXIIL*(1.0) and *synXIIL*(2.0)^29^. The colonies which were positive for 1.0 PCRtags but negative for 2.0 PCRtags indicated the specific loss of sequences on *synXIIL*.

### Tetrad analysis

Diploid strains were cultured in selective medium at 30 °C overnight. About 8 × 10^7^ cells were harvested and washed with ddH_2_O twice. Cells were resuspended with 50 μL 1 × Sporulation medium (10 g/L potassium acetate, 0.05 g/L zinc acetate dehydrate) and transferred into 2 ml 1 × Sporulation medium. The tubes were incubated at 25 °C for 3–10 days. Then cells were harvested and resuspended in 30 μL Zymolyase-100T (0.5 mg/ml Zymolyase-100T in 1 M sorbitol) for about 4–6 min at RT. 300 μL pre-cold ddH_2_O was added to stop digestion and 20 μL suspension was gently spread on YPD plates for further dissection under the microscope. These plates were cultured at 30 °C for appropriate days before imaging and replicated onto various selective media to identify their auxotroph and mating type.

### Analysis of protein length, genetic interaction, and gene ontology (GO)-terms

The theoretical protein length and genetic interaction information of each gene are queried through SGD YeastMine service (https://yeastmine.yeastgenome.org/yeastmine). Self-interactions of a gene were eliminated before calculation of GGI. The synthetic lethality is simultaneously inferred from the queries. The GO information of each gene is collected through R package org.Sc.sgd.db. Only the directly evidenced terms of each gene are represented in this package. The GO terms of all protein coding genes are inferred and summarized. The average number of GO terms for the genes on all chromosome arms are subsequently calculated.

### Assembly and screening of functional non-essential TUs

The pMV-*AmpR* plasmids, ORF plasmids and HCKan_terminator plasmids, hosting promoter pool (P), ORF (O) and terminator pool (T) were prepared for YeastFab assembly. The promoter fragments were amplified by ExTaq DNA polymerase (TaKaRa). The YeastFab assembly is performed according to the reaction system below: 1 μL 10X Buffer for T4 DNA Ligase (NEB), 0.1 μL Purified BSA 100X (Thermo), 0.2 μL T4 DNA Ligase (Thermo), 0.5 μL Esp3I (Thermo), 2 μL purified promoter PCR product, 2 μL ORF plasmid (20 ng/μL), 2 μL HCKan_terminator plasmid (20 ng/μL), 2 μL vector plasmid (20 ng/μL) and 0.2 μL ddH_2_O. Then carry out the following program in a thermal cycler: 37 °C for 2 h, 55 °C for 15 min, 80 °C for 15 min. The reaction products were transformed into *E. coli* DH5α and 5 mL LB liquid medium containing carbenicillin disodium (100 μg/mL) was added into the bacteria mix, shaking at 37 °C overnight. Plasmids were extracted from the bacteria mixture, transformed into yeast, and plated on selective plates.

Six individual colonies were randomly picked and cultured in selective liquid media overnight at 30 °C. The OD_600_ was measured, and the overnight cultures were diluted in sterile water to OD_600_ = 1. After four 10-fold gradient dilution, well-mixed cells were dropped onto indicated plates. The plates were cultured at appropriate temperature. The yeast strains with recovered phenotypes were selected. The plasmids carrying the synthetic TUs were isolated and identified by sanger sequencing.

### Flow cytometry analysis

Samples were selected at corresponding time points and cells were fixed with 70% ethanol overnight at 4 °C. Cells were resuspended in 50 mM sodium citrate (pH 7.0) and briefly sonicated on ice. Cells were resuspended in 50 mM sodium citrate (pH 7.0) and added with RnaseA (0.25 mg/mL) for incubation at 37 °C. Cells were washed with 50 mM sodium citrate (pH 7.0) and resuspended in 50 mM sodium citrate (pH 7.0) containing propidium iodide (16 µg/mL). The cells were incubated at room temperature for at least 1 h. Samples were proceeded with BD FACS Celesta for measurement. The software FlowJo was used for analysis and the fraction of cells at different stages was calculated with the Dean-Jett-Fox model.

### Microscope imaging

Cells were cultured in YPD overnight and subcultured into fresh YPD for several hours to get cells at log phase. Cells were harvested gently and washed once with ddH_2_O. Then cells were dropped on slides for further imaging with a Nikon A1 confocal microscope under 60× objective. To calculate cell size of strains at different stages with Image J, the cells of each strain were defined into two groups: G1 cells (not budding) and dividing cells (budding).

### Statistics and reproducibility

Error bars in this study represent SD. Unless specially noted, two-tailed *t* tests were used to compare different groups in this paper. All experiments were repeated independently at least three times. Differences were considered as statistically significant at *p* value < 0.05. * indicates *p* < 0.05, ** indicates *p* < 0.01, *** indicates *p* < 0.001, **** indicates *p* < 0.0001.

### Reporting summary

Further information on research design is available in the Nature Portfolio Reporting Summary linked to this article.

### Supplementary information

Supplementary information

Description of Additional Supplementary Files

Supplementary Data 1

Supplementary Data 2

Supplementary Data 3

Supplementary Data 4

Supplementary Data 5

Reporting Summary

Peer Review File

### Source data

Source data

## Data Availability

Nanopore sequencing data, RNA-seq data and Iso-seq data have been deposited at SRA with bioproject number PRJNA883530 Source data are provided with this paper.
