# Supplementary material for: Building a eukaryotic chromosome arm by de novo design and synthesis
Source: Nat Commun. 2023 Nov 30;14:7886. doi: 10.1038/s41467-023-43531-5 (PMC10689750; doi:10.1038/s41467-023-43531-5)

Supplementary information

Building a eukaryotic chromosome arm by *de novo* design and synthesis

Shuangying Jiang[1,9], Zhouqing Luo[1,2,9], Jie Wu[1], Kang Yu[1], Shijun Zhao [1,3], Zelin Cai[1,3], Wenfei Yu[1,3], Hui Wang[2], Li Cheng[1], Zhenzhen Liang[1,3], Hui Gao[1, 4], Marco Monti[5], Daniel Schindler[5], Linsen Huang[1,3], Cheng Zeng[1], Weimin Zhang[6], Chun Zhou[1,3], Yuanwei Tang[1], Tianyi Li[1], Yingxin Ma[1], Yizhi Cai[1,5], Jef D. Boeke[6,7], Qiao Zhao[1,*] Junbiao Dai[1,3,8,*]

Affiliations:

[1]CAS Key Laboratory of Quantitative Engineering Biology, Guangdong Provincial Key Laboratory of Synthetic Genomics and Shenzhen Key Laboratory of Synthetic Genomics. Shenzhen Institute of Synthetic Biology, Shenzhen Institute of Advanced Technology, Chinese Academy of Sciences, Shenzhen, China.
[2]State Key Laboratory of Cellular Stress Biology, Innovation Center for Cell Signaling Network, School of Life Sciences, Faculty of Medicine and Life Sciences, Xiamen University, Xiamen, Fujian 361102, China
[3]University of Chinese Academy of Sciences, Beijing, China.
[4]Institute of Molecular Physiology, Shenzhen Bay Laboratory, Shenzhen, China
[5]Manchester Institute of Biotechnology, University of Manchester, Manchester M1 7DN, U.K.
[6]Institute for Systems Genetics and Department of Biochemistry and Molecular Pharmacology, NYU Langone Health, New York, NY 10016 USA
[7]Department of Biomedical Engineering, NYU Tandon School of Engineering, Brooklyn, NY 11201 USA
[8]Shenzhen Branch, Guangdong Laboratory for Lingnan Modern Agriculture, Key Laboratory of Synthetic Biology, Ministry of Agriculture and Rural Affairs, Agricultural Genomics Institute at Shenzhen, Chinese Academy of Agricultural Sciences, Shenzhen, China
[9]These authors contributed equally
* Correspondence to: daijunbiao@caas.cn or qiao.zhao@siat.ac.cn

Supplementary Figures 1-9
Supplementary Table 1

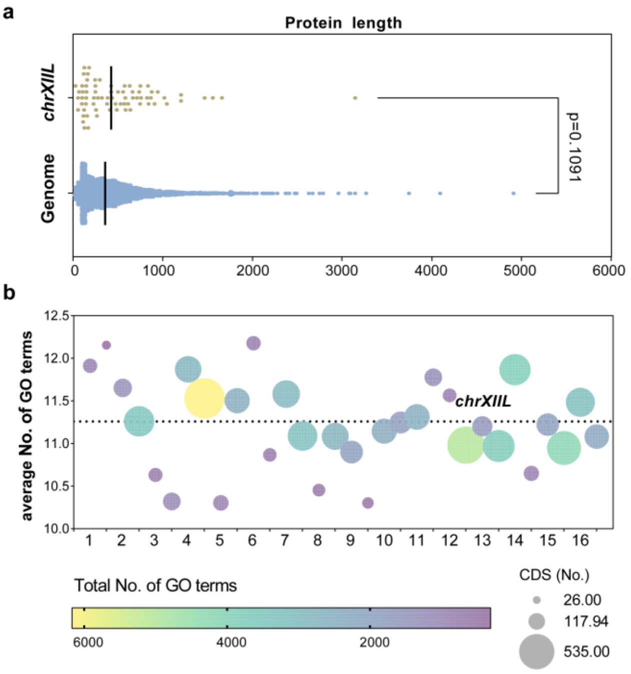

Supplementary Fig.1 Analysis of the protein length and function complexity of the genes on *chrXIIL.*

a, Theoretical protein length of the genes on *chrXIIL* (73 genes) or all genes in yeast genome (6713 genes). Data is from SGD database. Black line: the median length (427 amino acids for the genes on *chrXIIL* and 359 amino acids for all genes in yeast genome). Unpaired t-test (two-tail) was used compare the two groups. Source data are provided as a Source Data file. **b**, The average directly evidenced GO terms of genes in all 32 chromosome arms. Dashed line indicates genome-wide average number of GO terms per gene. The color and size of the bubbles represent total number of GO terms and CDSs of every chromosome arm, respectively. *chrXIIL* is labeled.

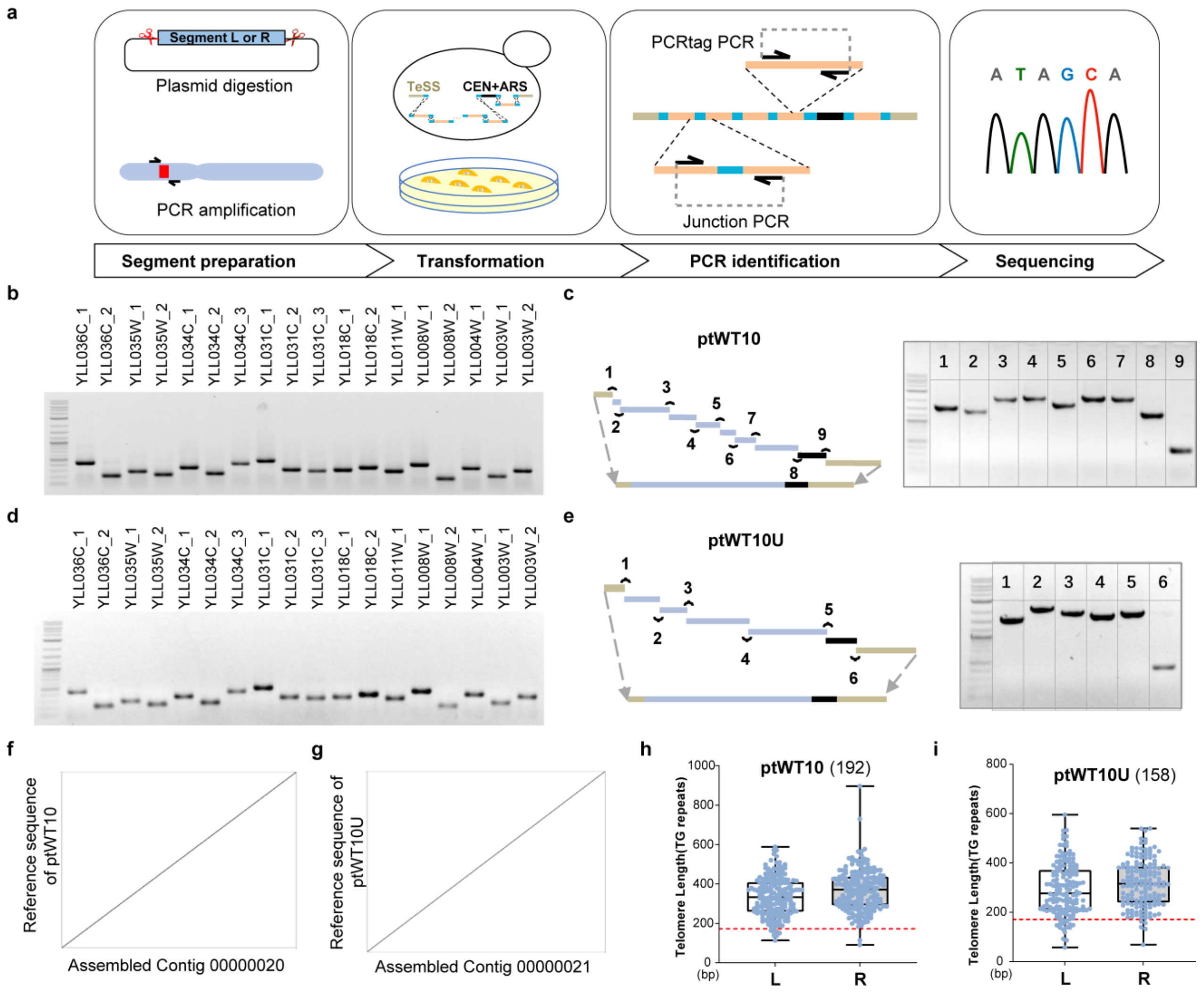

Supplementary Fig.2 Construction of neochromosomes harboring essential genes from *chrXIIL*.
a, The schematic workflow of transformation-assisted linear chromosome construction. **b**, Validation of ptWT10 neochromosome by PCRTag analysis. **c**, Confirmation of assembled ptWT10 by junction analysis. The two ends and the black fragment represent segment L, R and M as in Fig.1a. **d**, Validation of ptWT10U neochromosome. Similar analysis as in (**b**). **e**, Confirmation of ptWT10U. Similar analysis as **c**. **f-g**, Dot plot analysis after whole genome sequencing showed the correct assembly of ptWT10 and ptWT10U. **h-i**, Length analysis of telomere TG repeats in neochromsomes. The bounds of the box were the upper and lower quartile with the median value in the center. The whiskers indicated the minimum and maximum. The red dashed line indicates the length of synthesized TG repeats. Only sequencing reads covering the entire neochromosomes (n=192 for ptWT10 and n=158 for ptWT10U) were used for calculation. Source data are provided as a Source Data file.

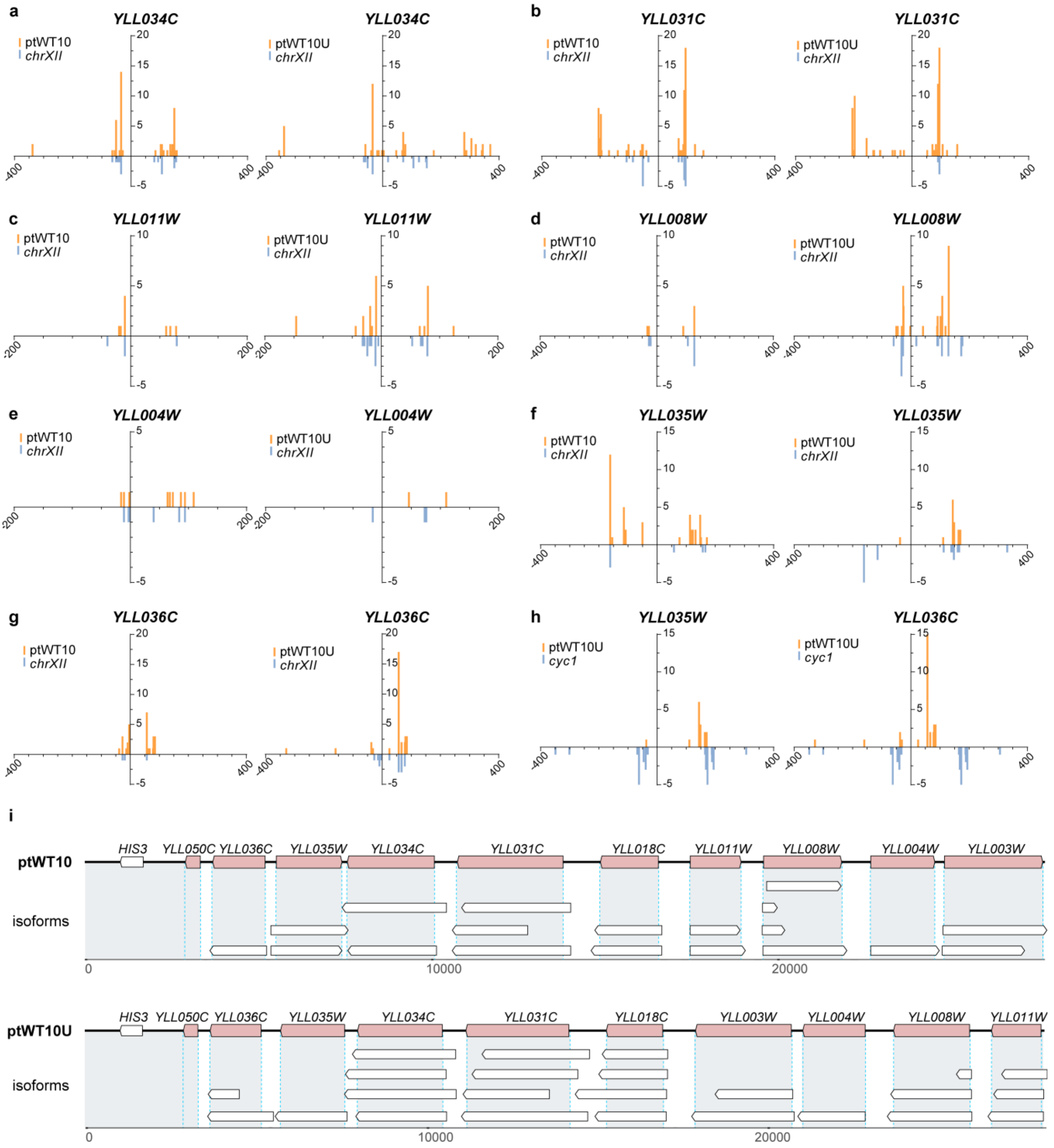

Supplementary Fig.3 Transcription analysis of genes on ptWT10 and ptWT10U.

a-b, The distribution of TSS and TES for the two genes, *YLL034C* and *YLL031C*, with the same direction on ptWT10 and ptWT10U. **c-e**, The distribution of TSS and TES for other three genes, *YLL011W*, *YLL008W* and *YLL004W*, with different directions on ptWT10 and ptWT10U. **f-g**, The distribution of TSS and TES for the two genes, *YLL035W* and *YLL036C*, with different regulation sequences on ptWT10 and ptWT10U. **h**, Comparison of the distribution of TSS/TES of *YLL035W* and *YLL036C* on the ptWT10U to native *CYC1* gene. **i**, The isoforms identified after Iso-seq collapse for the genes on ptWT10 and ptWT10U (except *YLL050C*) were exhibited. None of the isoforms went through more than two open reading frames (ORFs).

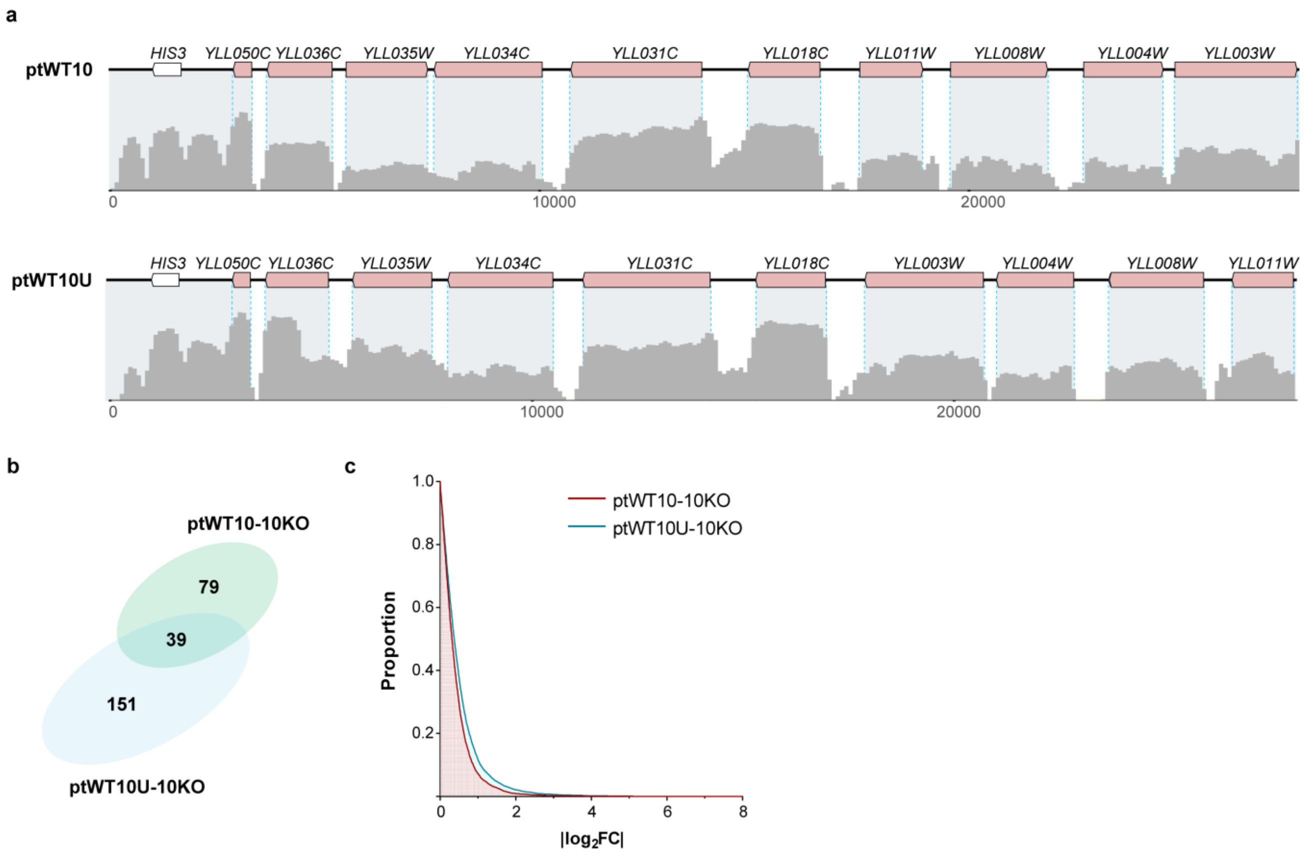

Supplementary Fig.4 RNA-seq results of ptWT10-10KO and ptWT10U-10KO.

a, Transcription expression of the essential genes on ptWT10 and ptWT10U in the ptWT10-10KO and ptWT10U-10KO strains. Blue dashed lines, the boundaries of CDSs. Y axis means $\log_{10}$ (average RNA sequencing depth of neochromosomes +1). **b**, Venny diagram of other differentially expressed genes in ptWT10-10KO and ptWT10U-10KO.**c**, Transcriptome-wide perturbation of ptWT10-10KO and ptWT10U-10KO. BY4742 was used as the control for normalization. X axis means $|\log_2 FC|$. Y axis means the percentage of genes with $|\log_2 FC|>$ the value of X axis.

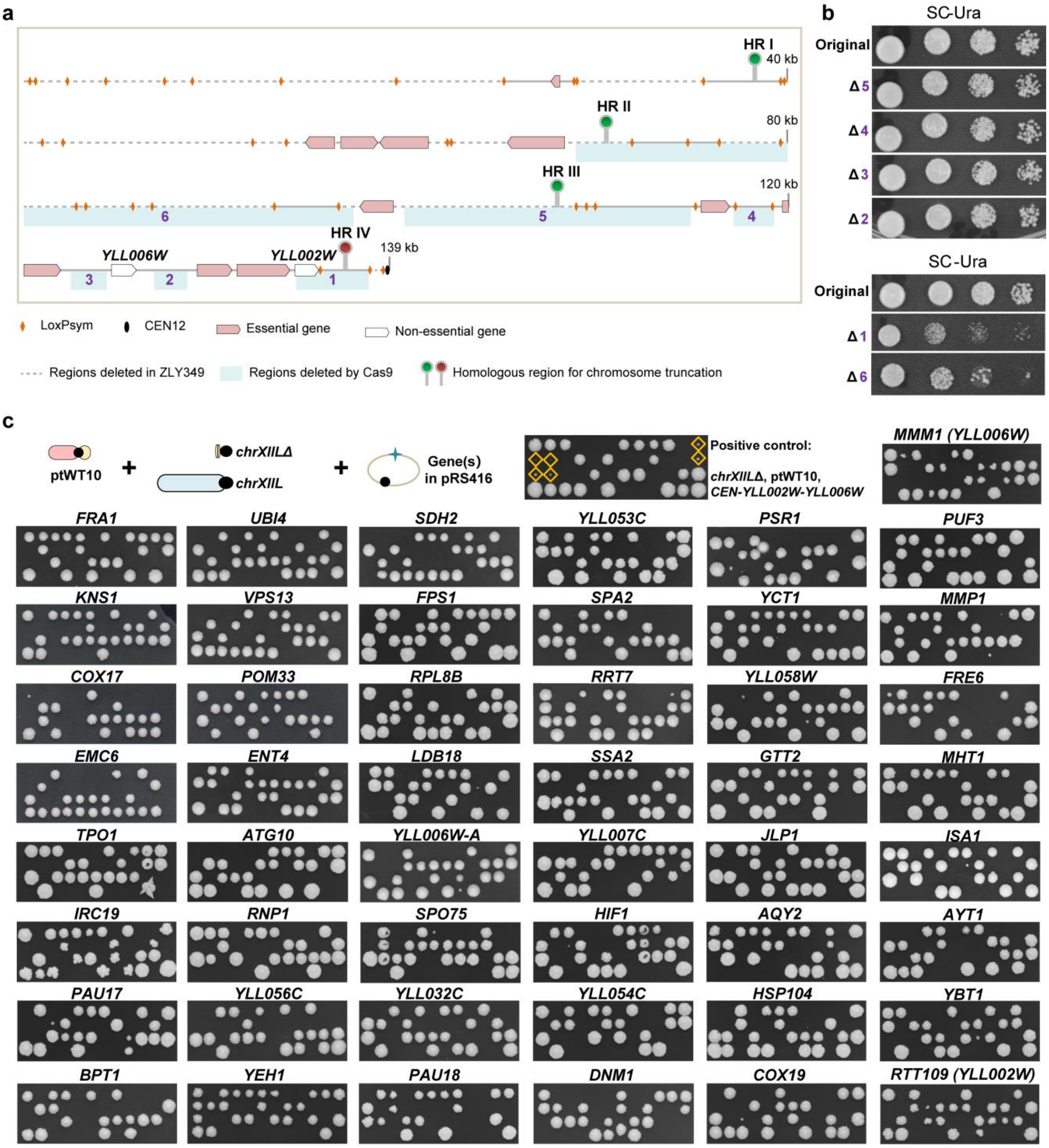

Supplementary Fig.5 Simplification of *chrXIIL*.

a, The schematic diagram of left arm of *synXII*. Balloons (HR I-IV), positions of homologous regions used for chromosome truncation in Fig.2. **b**, The growth of strains with corresponding regions deleted in (**a**). **c**, None of the 49 groups of 11-gene combination could support cell viability. The growth of dissected spores on YPD after at least 7-day culture at 30°C was showed. These 49 non-essential genes were cloned into pRS416 for expression. The length of promoters and terminators were about 500bp and 200bp, respectively. These expression vectors were transformed into the hetero-diploid strains containing the linear neochromosome with 10 essential genes (*chrXIIL*/*chrXIILΔ* ptWT10). At least two different transformants of each gene were proceeded for sporulation and tetrad analysis. In the positive control (*YLL002W*+*YLL006W* in pRS416), four viable spores were observed, and the small ones were verified to be the correct haploid strains——*chrXIILΔ*, ptWT10, *CEN-YLL002W-YLL006W*. Any tetrads with more than three spores were analyzed, such as the spores for *YEH1*, but these strains were either diploid or haploid with the wrong genotypes.

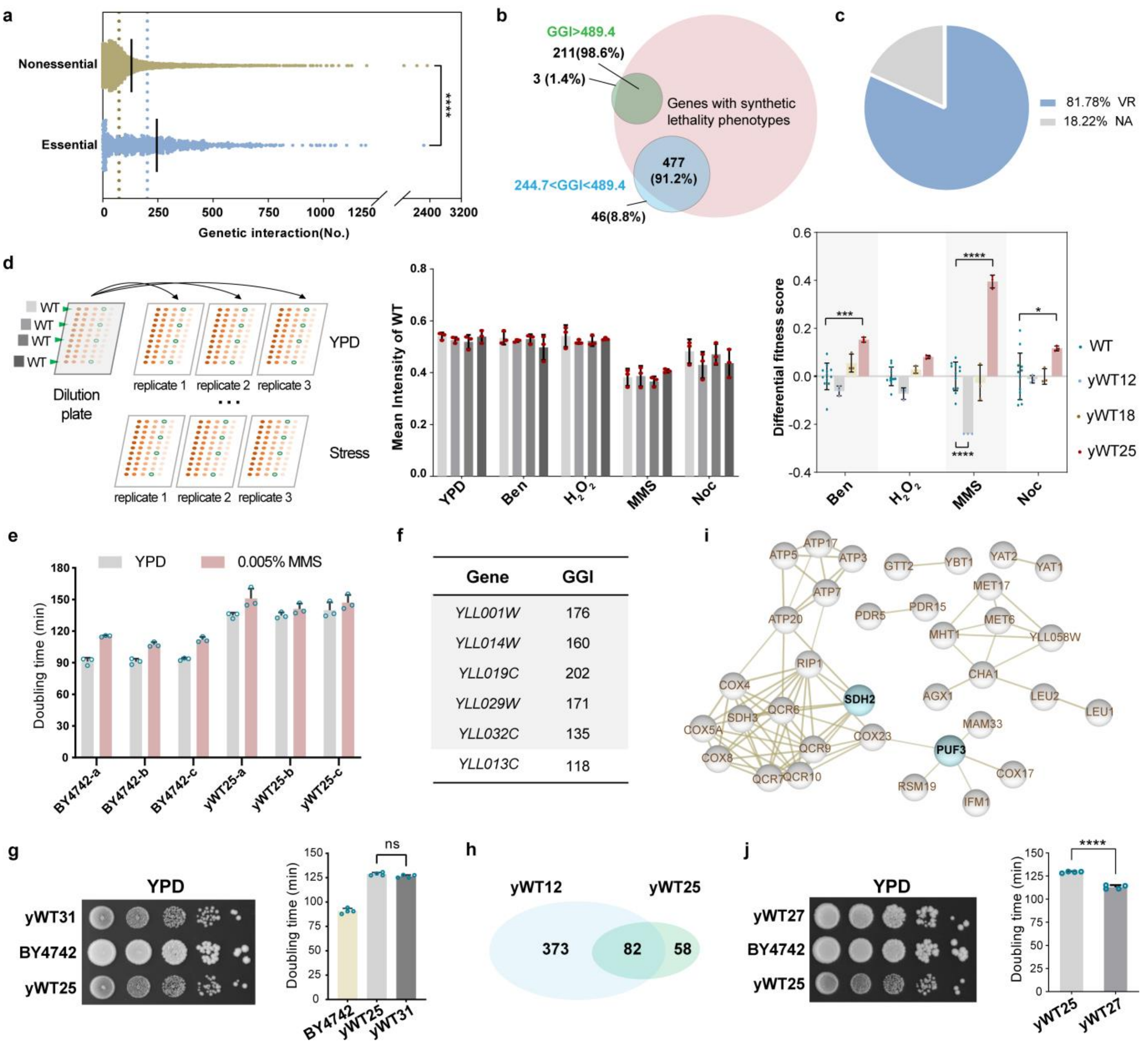

Supplementary Fig.6 Construction and analysis of strains with different neochromosomes.

a, Genetic interaction number of all genes (1054 essential genes and 4943 non-essential genes) in yeast genome. Data is from SGD database. Genetic interaction number of certain gene means the number of other genes that exhibit genetic interaction with it. Black line: the average genetic interaction number (244.7 for essential genes and 129.3 for non-essential genes). Dash line: the median genetic interaction number (brown: 73 for non-essential genes; blue: 202 for essential genes). Unpaired t-test (two-tail) was used compare the two groups, p=1.66E-54. Source data are provided as a Source Data file. **b**, Venn diagram of genes with synthetic lethality phenotypes in yeast genome (3579 genes), genes with GGI>489.4 (green) and genes with 244.7<GGI<489.4 (blue). **c**, More than 80% genes with GGI>489.4 exhibited obvious growth defects when deleted individually. VR: genes showed decreased vegetative growth or absent respiratory growth at 30℃ when deleted. NA: other genes. **d**, Differential fitness scores of the strains in different conditions. Positive differential fitness scores indicate a better resistance to a stress condition than WT(BY4741), and negative differential fitness scores indicate fitness defects in the corresponding condition. The "Mean Intensity" value of the spots in the fourth gradient was used as a measure of cell growth[1,2]. No significant growth difference was detected among BY4741 cells (the spots marked with green circles) located at different positions. The average "Mean Intensity" for the four replicates of wild type cells at different positions on the same plate in each condition were normalized to be 1. The differential fitness score for each strain in each condition was calculated as before[3]. The 2way ANOVA analysis was used to calculate the difference significance. *P<0.05, **P<0.01, ***P<0.001, ****P<0.0001. Source data are provided as a Source Data file. **e,** Doubling time of strains.

The growth of cells from three different colonies of BY4742 and yWT25 in different medium at 30°C were analyzed, and three technical repeats were conducted for each colony. The doubling times were used to calculate logarithmic strain coefficients (LSC) and logarithmic phenotype index (LPI) [4,5]of strains in Fig.3d. Source data are provided as a Source Data file. **f,** The six genes used to construct ptWT31 and yWT31. **g,** The growth of strains in rich medium at 30°C. Four technical repeats were conducted for each strain to calculate the doubling time. Unpaired t-test (two-tail) was used compare the two groups. ns: no significance, p=0.0727. Source data are provided as a Source Data file. **h,** Comparison of differentially expressed genes in yWT12 and yWT25. **i,** *SDH2* and *PUF3* were selected to construct ptWT27 and yWT27. The protein-protein interaction network was generated with the string database (Szklarczyk et al., 2021) (experiment and database, cutoff score is 0.7) using the 82 shared genes in (**h**). **j,** The growth of strains in rich medium were analyzed as in (**g**). Unpaired t-test (two-tail) was used compare the two groups, p=9.03E-06. Source data are provided as a Source Data file.

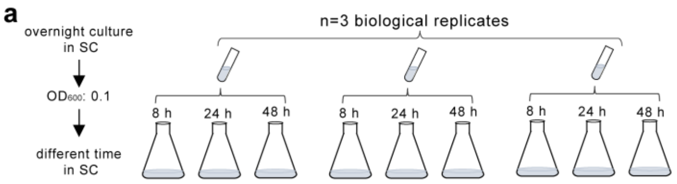

OD600	8 h			24 h			48 h		
BY4742	3.30	3.65	3.65	7.70	7.40	7.60	9.80	9.60	9.80
yWT12	0.29	0.29	0.29	3.10	3.10	3.30	4.60	5.00	4.80
yWT25	1.06	1.04	1.14	6.80	6.70	6.80	7.40	7.20	6.80

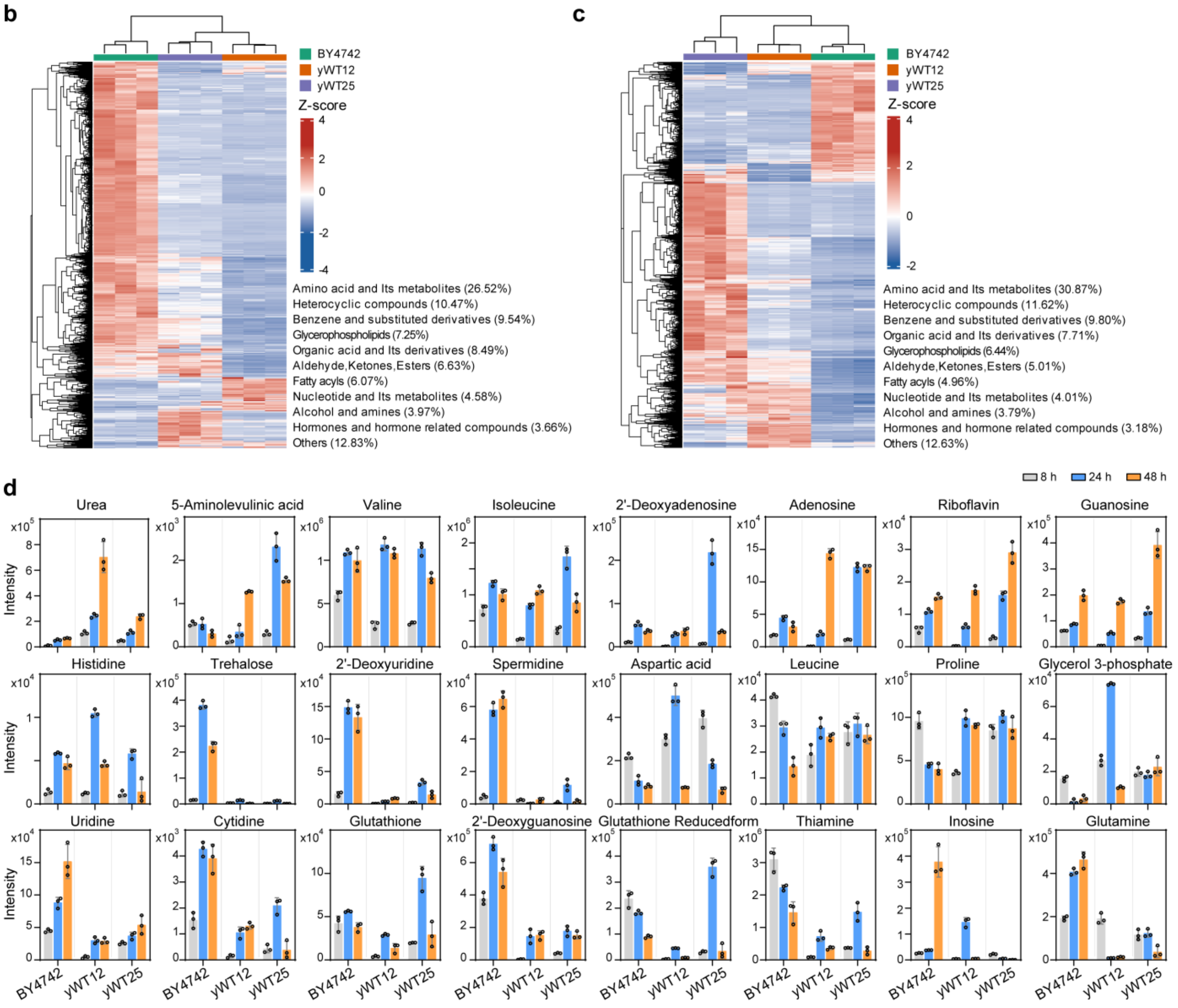

Supplementary Fig.7 BY4742, yWT12 and yWT25 exhibit distinct metabolic profiles at different growth phases.

a, Cells with different growth times in SC medium (8 h, 24 h and 48 h) were used for untargeted metabolomics analysis. **b-c,** Heatmap of DMs based on the one-way ANOVA ($p < 0.05$ and $|log_2FC| > 1$) and the proportion of different types of DMs in BY4742, yWT12, and yWT25 at 8 h (**b**) and 48 h (**c**). **d,** Other DMs belonging to the ABC transporters-dependent pathway were assessed. Data are shown as Fig. 4g. Source data are provided as a Source Data file.

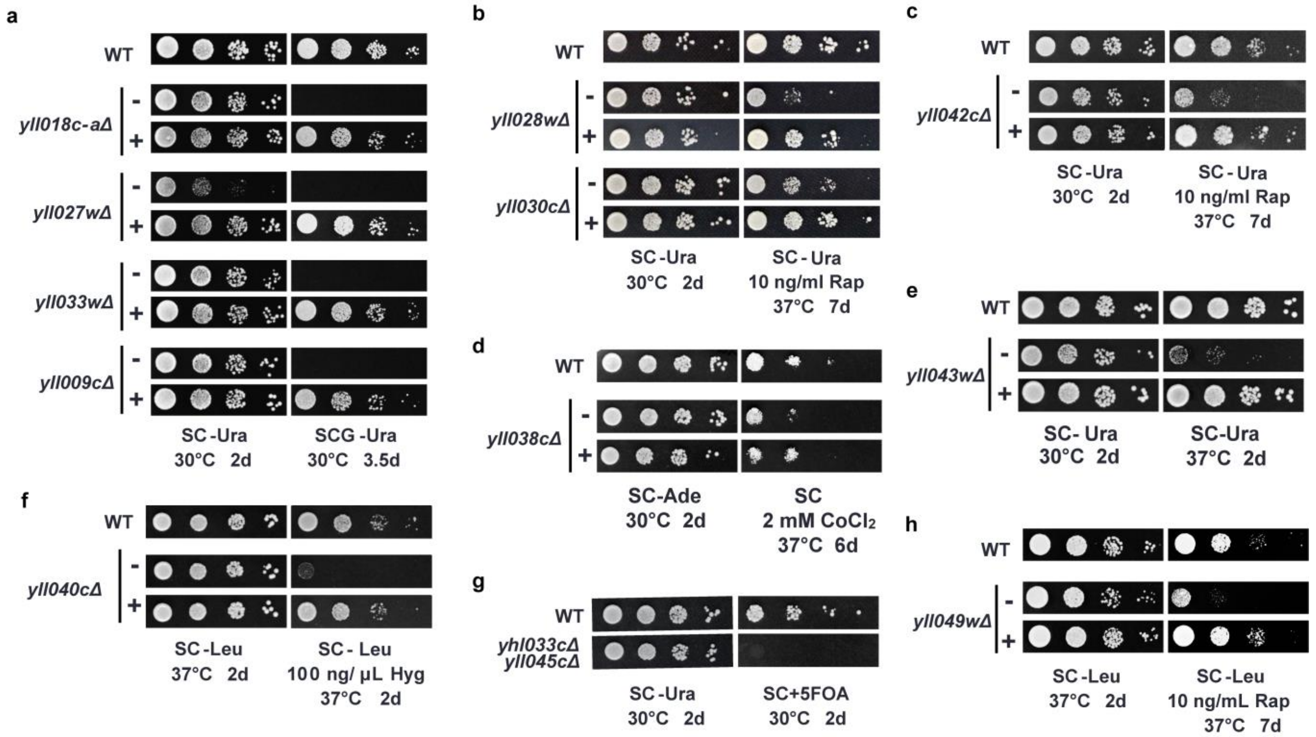

Supplementary Fig.8 Reconstructing functional non-essential genes with artificial sequences.
a-h, Serial dilution assay to measure the function of reconstructed nonessential genes. SCG, synthetic complete media with 3% glycerol. Hyg, hygromycin. In **d**, *yll038c* deletion was reported to be sensitive to $CoCl_2$ [6]. The limited sensitivity was rescued by the addition of reconstructed *syn038c*. In **g**, *yhl033c* and *yll045c* double deletion is lethal [7]. WT, BY4741/2 with empty vector; -, empty vector; +, with refactored gene.

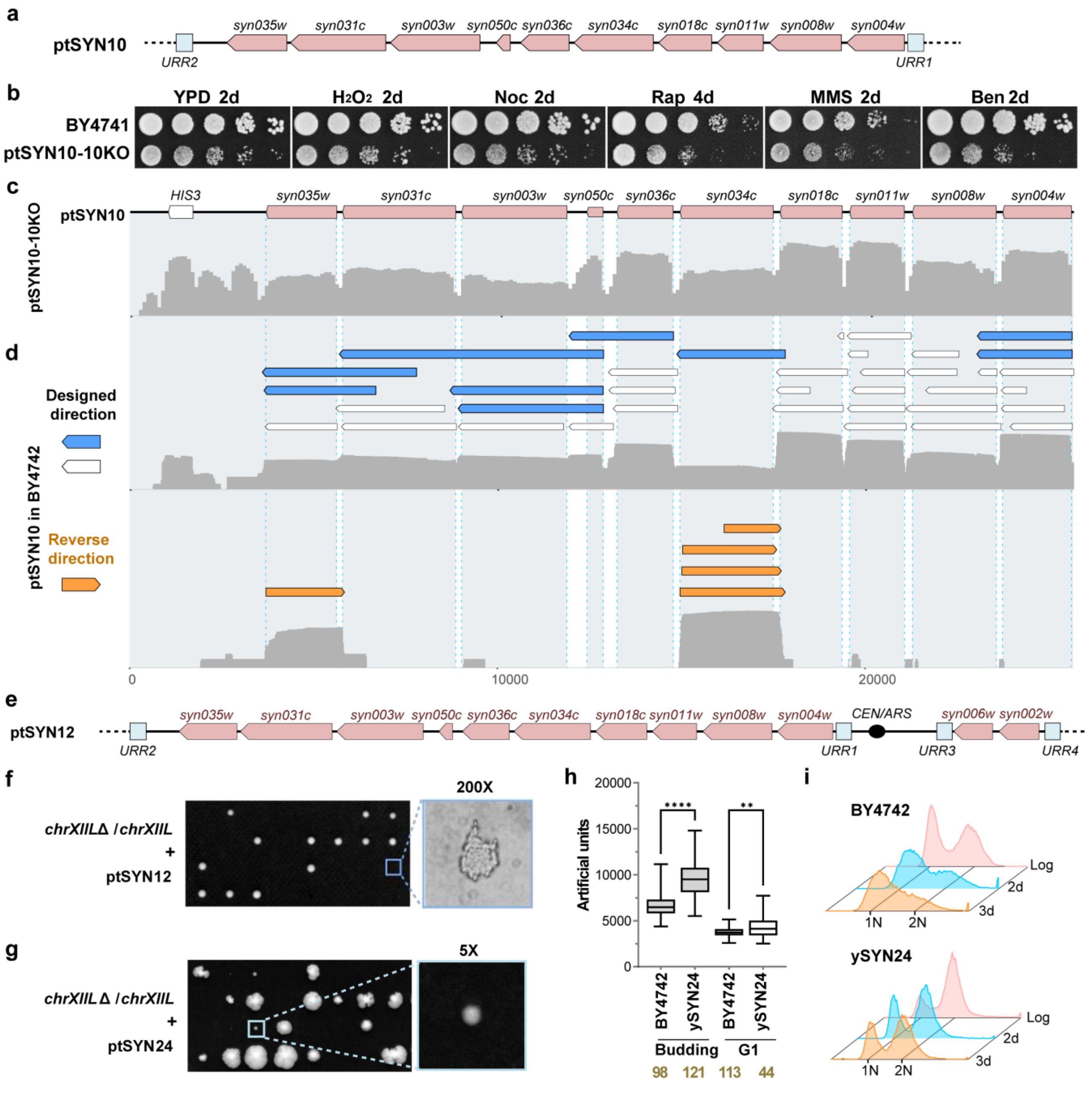

Supplementary Fig.9 Construction of the neochromosomes with reconstructed genes.

a,The schematic diagram of the reconstructed TUs added in ptSYN10. **b**, Fitness analysis of the strain ptSYN10-10KO. **c**, Expression profile of genes from ptSYN10 in ptSYN10-10KO. The RNA-seq data was shown in the same way as Supplementary Fig.4a. **d**, The Iso-seq results of the ptSYN10 in BY4742. The transcripts of each gene on ptSYN10 were identified and divided into two categories: transcripts in designed direction and the ones in reverse direction. The identified isoforms for the synthetic genes were exhibited: the ones which went through more than two ORFs (blue), the anti-sense isoforms (orange) and others (white). **e**, The schematic diagram of reconstructed TUs in ptSYN12. **f-g**, Tetrad analysis of *chrXIILΔ/chrXIIL* heterozygous strains containing ptSYN12 or ptSYN24. **h**, Quantification of cell size of ySYN24 strain. The box plot (min-max) shows cell size of budding cells and G1 cells (a single cell without bud). Cell sizes were measured by ImageJ. The number of cells counted for each strain were marked in brown. The bounds of the box were the upper and lower quartile with the median value in the center. The whiskers indicated the minimum and maximum. Unpaired t-test (two-tail) was used compare two groups. For budding cells, p=2.93E-31; for G1 cells, p=0.002845. Source data are provided as a Source Data file. **i**, FACS analysis of cells arrested by nutrient depletion. Cells in log phase were used as the control.

Supplementary Table 1. The information used to identify the nonessential gene set.

Non-essential gene in *chrXIIL*	Gene deleted in the strain Δ1	Gene in region IV	Affecting vegetative or respiratory growth [b]	Synthetic lethality number [b]	GGI [a]	Gene remained in ZLY348	Gene selected in yWT25
**YLL002W**	+	+	**V**	**29**	**567**	+	+
YLL001W	+	+		2	176	+	
**YLL006W**		+	**VR**	**9 (YLL040C)**	**602**	+	+
**YLL009C**		+	**R**	2	198	+	+
YLL014W		+			160	+	
YLL010C		+		2	121	+	
YLL013C		+		1	118	+	
YLL006W-A		+			84	+	
YLL005C		+			80	+	
YLL007C		+			80	+	
YLL012W		+			40	+	
**YLL049W**				**11**	**443**	+	+
**YLL039C**			**VR**	**2**	**372**	+	+
**YLL043W**			**V**	**5**	**352**	+	+
**YLL040C**			**R**	**2 (YLL006W)**	**326**		+
**YLL045C**			**V**	**1**	**272**	+	+
**YLL038C**			**V**	1	192	+	+
**YLL042C**			**VR**		133	+	+
**YLL033W**			**VR**		122	+	+
**YLL028W**			**V**		66	+	+
**YLL027W**			**VR**	3	61	+	+
**YLL018C-A**			**VR**	1	38	+	+
**YLL030C**			**V**		--	+	+
YLL041C			R	1	145		
YLL023C				2	207		
YLL019C				1	202	+	
YLL029W				2	171	+	
YLL021W				17	144		
YLL032C					135	+	
YLL015W					104	+	
YLL053C				1	95		
YLL024C				4	94		
YLL046C					82		
YLL026W				1	70		
YLL048C				1	57		
YLL056C					48		
YLL058W					46		
YLL055W					44		
YLL057C					44		
YLL054C					40		
YLL063C				1	27		
YLL061W					25		
YLL025W					25	+	
YLL060C				1	26		
YLL022C					23		
YLL051C					23	+	
YLL062C					15		
YLL052C					9		
YLL064C					--	+	
YLL019W-A[c]					--		
YLL020C[c]					--	+	
YLL037W[c]					--	+	
YLL044W[c]					--	+	
YLL047W[c]					--		
YLL059C[c]					--		
YLL065W[c]					--		
YLL066W-A[c]					--		
YLL067W-A[c]					--		
YLL017W[d]					25	+	
YLL016W[d]					24	+	
YLL066W-B[e]					69		
YLL066C[e]					--		
YLL067C[e]					--		

[a] GGI: the number of genes exhibiting genetic interaction with the indicated gene. Data is from SGD: https://www.yeastgenome.org.

[b] Synthetic lethality number: the number of genes exhibiting synthetic lethality with the indicated gene.

[c] Dubious genes, [d] pseudogene, [e] genes located at telomere, R: absent respiratory growth at 30°C when deleted, V: decreased vegetative growth when deleted

1 Lamprecht, M. R., Sabatini, D. M. & Carpenter, A. E. CellProfiler: free, versatile software for automated biological image analysis. *Biotechniques* **42**, 71-75, doi:10.2144/000112257 (2007).

2 Lawless, C., Wilkinson, D. J., Young, A., Addinall, S. G. & Lydall, D. A. Colonyzer: automated quantification of micro-organism growth characteristics on solid agar. *BMC Bioinformatics* **11**, 287, doi:10.1186/1471-2105-11-287 (2010).

3 Costanzo, M. *et al.* Environmental robustness of the global yeast genetic interaction network. *Science* **372**, doi:10.1126/science.abf8424 (2021).

4 Warringer, J., Ericson, E., Fernandez, L., Nerman, O. & Blomberg, A. High-resolution yeast phenomics resolves different physiological features in the saline response. *Proc Natl Acad Sci U S A* **100**, 15724-15729, doi:10.1073/pnas.2435976100 (2003).

5 Lin, Y., Zou, X., Zheng, Y., Cai, Y. & Dai, J. Improving Chromosome Synthesis with a Semiquantitative Phenotypic Assay and Refined Assembly Strategy. *ACS Synth Biol* **8**, 2203-2211, doi:10.1021/acssynbio.8b00505 (2019).

6 Zhao, Y. Y. *et al.* Genetic analysis of oxidative and endoplasmic reticulum stress responses induced by cobalt toxicity in budding yeast. *Biochim Biophys Acta Gen Subj* **1864**, 129516, doi:10.1016/j.bbagen.2020.129516 (2020).

7 Steffen, K. K. *et al.* Ribosome deficiency protects against ER stress in Saccharomyces cerevisiae. *Genetics* **191**, 107-118, doi:10.1534/genetics.111.136549 (2012).

Source data:

Supplementary Fig.2b

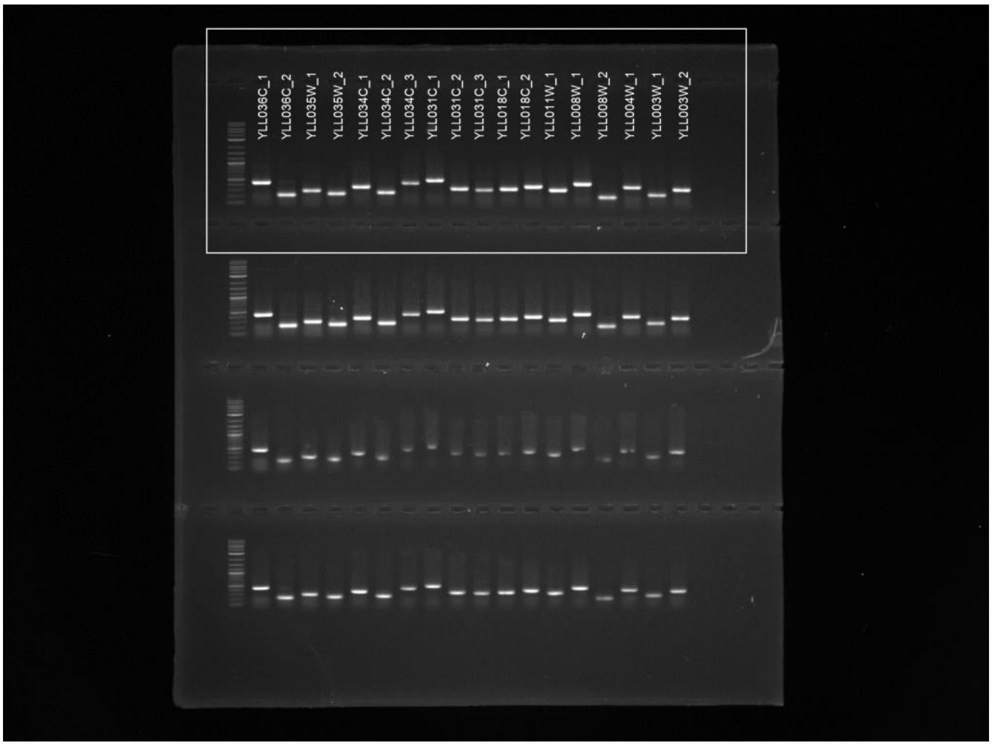

Supplementary Fig.2c

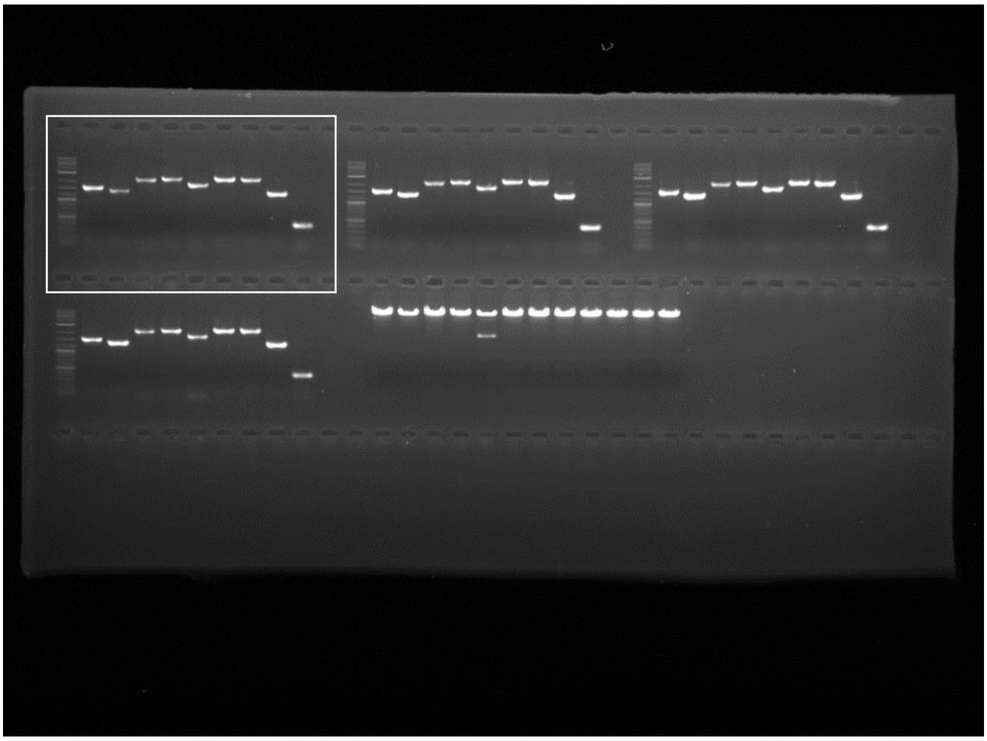

Supplementary Fig.2d

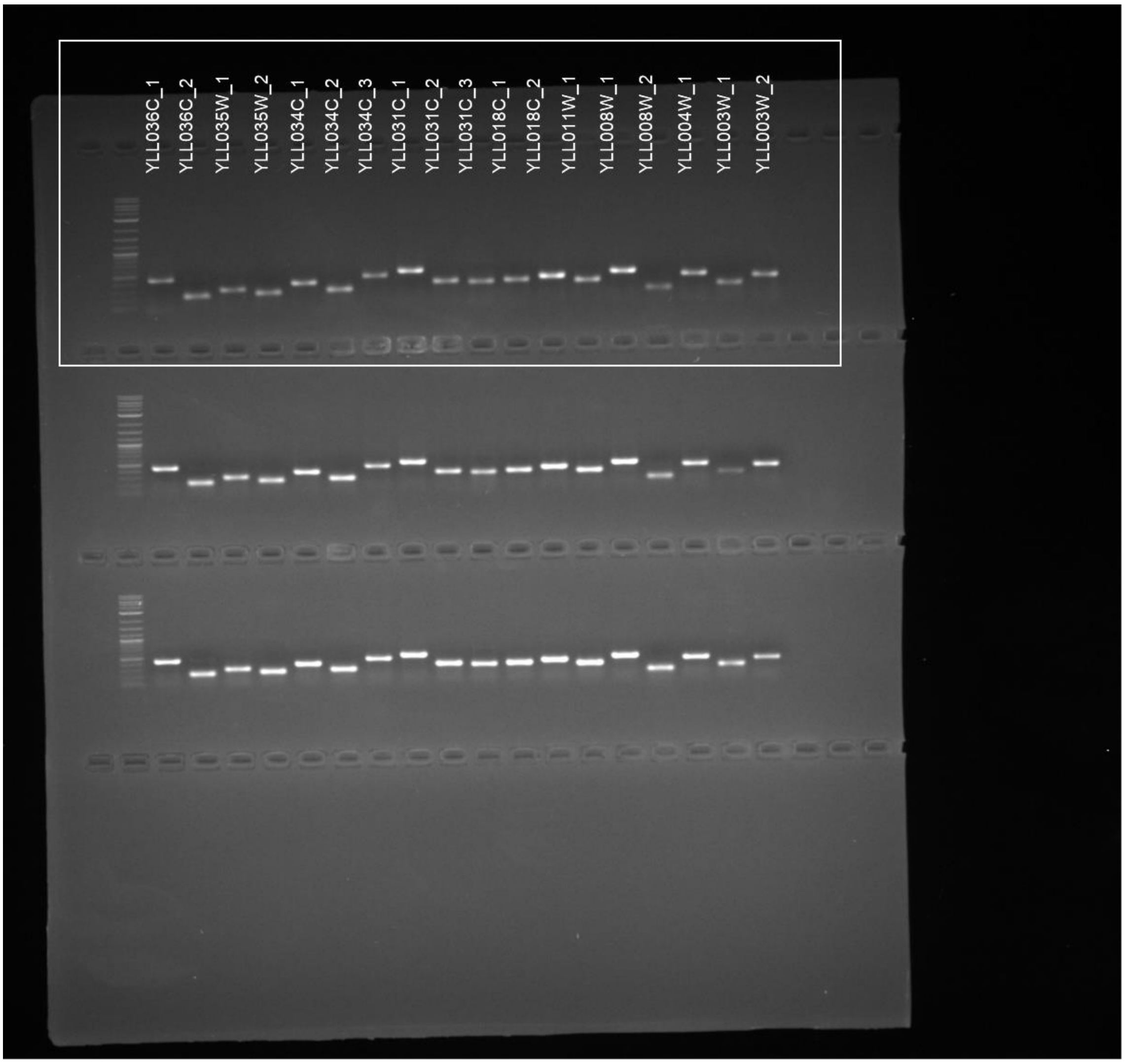

Supplementary Fig.2e

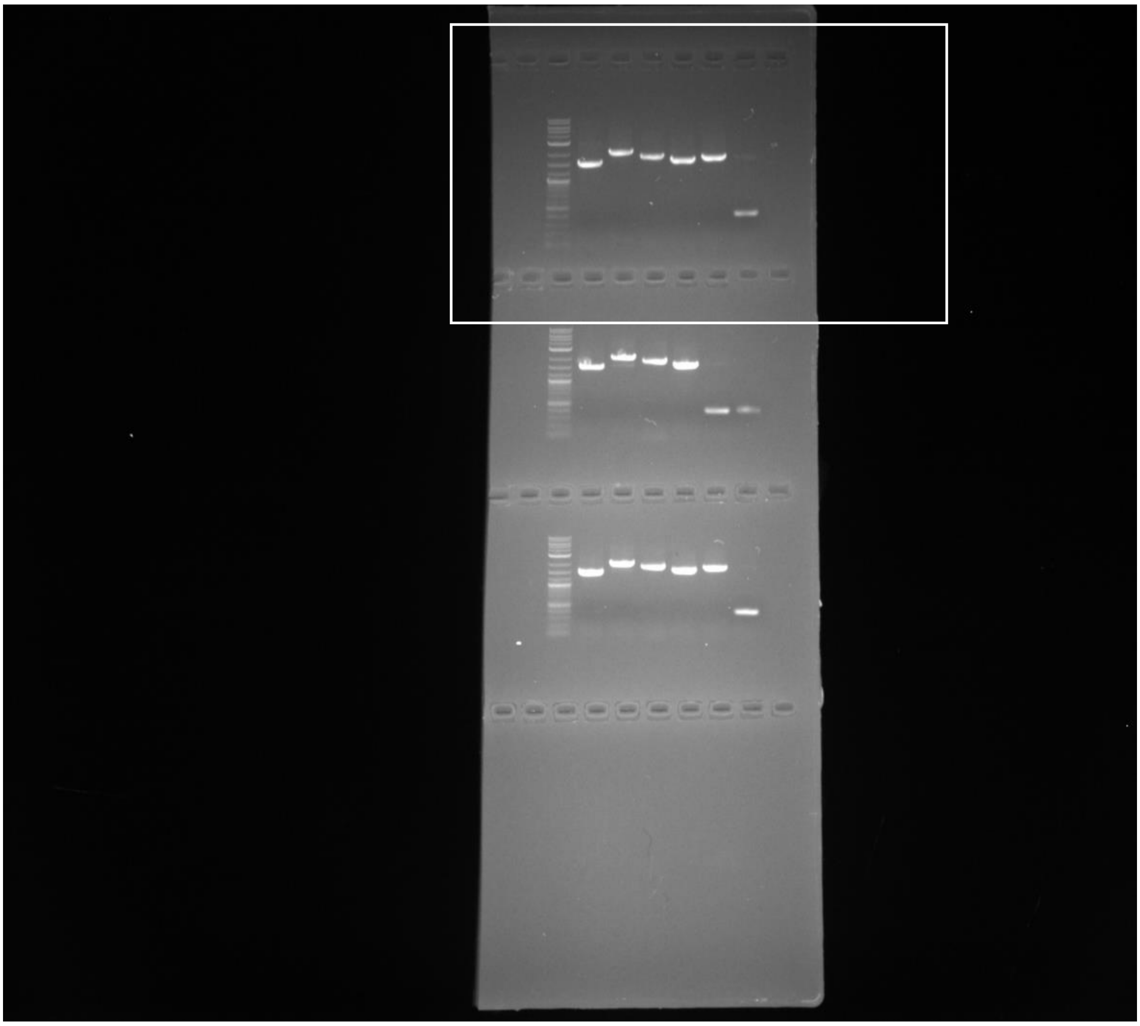

Supplement: Supplementary file 1 — Supplementary information [file 41467_2023_43531_MOESM1_ESM.pdf]
