# Supplementary material for: Building a eukaryotic chromosome arm by de novo design and synthesis
Source: Nat Commun. 2023 Nov 30;14:7886. doi: 10.1038/s41467-023-43531-5 (PMC10689750; doi:10.1038/s41467-023-43531-5)
Supplement: Supplementary file 2 — Description of Additional Supplementary Files [file 41467_2023_43531_MOESM2_ESM.pdf]

Title: Supplementary Data 1.
Legends: The designed sequences of neo-chromosomes and the identified variations of assembled neo-chromosomes.

Title: Supplementary Data 2.
Legends: Differentially expressed genes (DEG) in corresponding strains, including ptWT10-10KO, ptWT10U-10KO, yWT12, yWT25 and ySYN24. DESeq2 was used to calculate Benjamini-Hochberg adjusted p-values and fold change (FC) values. Differential expression in this study was defined as $|log_2FC| > 1$ and $-log_{10}$(Adjusted p-value) >4. BY4742 was used as the control strain.

Title: Supplementary Data 3.
Legends: Sequences of reconstructed TUs, synthetic promoters and synthetic terminators.

Title: Supplementary Data 4.
Legends: Strains used in this study.

Title: Supplementary Data 5.
Legends: Oligonucleotides used in this study.
