# Supplementary material for: Building a eukaryotic chromosome arm by de novo design and synthesis
Source: Nat Commun. 2023 Nov 30;14:7886. doi: 10.1038/s41467-023-43531-5 (PMC10689750; doi:10.1038/s41467-023-43531-5)
Supplement: Supplementary file 8 — Reporting Summary [file 41467_2023_43531_MOESM8_ESM.pdf]

Corresponding author(s): Junbiao Dai
Last updated by author(s): Nov 7, 2023

# Reporting Summary

Nature Portfolio wishes to improve the reproducibility of the work that we publish. This form provides structure for consistency and transparency in reporting. For further information on Nature Portfolio policies, see our Editorial Policies and the Editorial Policy Checklist.

## Statistics

For all statistical analyses, confirm that the following items are present in the figure legend, table legend, main text, or Methods section.

| n/a | Confirmed | |
|---|---|---|
| ☐ | ☒ | The exact sample size (*n*) for each experimental group/condition, given as a discrete number and unit of measurement |
| ☐ | ☒ | A statement on whether measurements were taken from distinct samples or whether the same sample was measured repeatedly |
| ☐ | ☒ | The statistical test(s) used AND whether they are one- or two-sided<br>*Only common tests should be described solely by name; describe more complex techniques in the Methods section.* |
| ☒ | ☐ | A description of all covariates tested |
| ☒ | ☐ | A description of any assumptions or corrections, such as tests of normality and adjustment for multiple comparisons |
| ☐ | ☒ | A full description of the statistical parameters including central tendency (e.g. means) or other basic estimates (e.g. regression coefficient) AND variation (e.g. standard deviation) or associated estimates of uncertainty (e.g. confidence intervals) |
| ☐ | ☒ | For null hypothesis testing, the test statistic (e.g. *F*, *t*, *r*) with confidence intervals, effect sizes, degrees of freedom and *P* value noted<br>*Give P values as exact values whenever suitable.* |
| ☒ | ☐ | For Bayesian analysis, information on the choice of priors and Markov chain Monte Carlo settings |
| ☒ | ☐ | For hierarchical and complex designs, identification of the appropriate level for tests and full reporting of outcomes |
| ☐ | ☒ | Estimates of effect sizes (e.g. Cohen's *d*, Pearson's *r*), indicating how they were calculated |

*Our web collection on statistics for biologists contains articles on many of the points above.*

## Software and code

Policy information about availability of computer code

| | |
|---|---|
| Data collection | Nanopore sequencing was performed using the MinION platform with FLO-MIN106D for a 72-h run. Base calling, adaptor removal, and low-quality base filtering based on fast5 files were done by ONT software MinKNOW.<br>For the stability of neochromosomes,300-1000 bp libraries were prepared using the Nextera DNA Flex Library Prep Kit for purified genomic DNA and then sequenced on the Illumina Miseq platform with PE250 strategy.<br>The non-stranded RNA sequencing libraries were prepared and sequenced using Next® UltraTM RNA Library Prep Kit for Illumina®.<br>The full-length transcriptomes of indicated strains were sequenced using the PacBio platform.<br>For untargeted metabolomic analysis, the MS data acquisition was operated using the information-dependent acquisition (IDA) mode using Analyst TF 1.7.1 Software (Sciex, Concord, ON, Canada).<br>For FACS, fixed cell samples were proceeded with BD FACS Celesta for measurement.<br>For doubling time assay, cells was cultivated in an Epoch2 microplate photometer (BioTek) at 30°C for 36-48 h in a selected medium.<br>For PFGE, plug samples were resolved on a 1% agarose gel in 0.5 X TBE for 16 hours at 14°C on a BioRad CHEF Mapper XA Pulsed Field Electrophoresis System. The BioRad ChemiDoc MP Imaging System was used for imaging of gels and blots.<br>For microscope imaging, cells were dropped on slides for further imaging with a Nikon A1 confocal microscope. |
| Data analysis | Nanopore sequencing data: The FastQ files were filtered by NanoFilt to remove short (read length < 500) and low-quality (average read quality score < 7) reads. The remaining reads were mapped to reference genome by NGMLR and assembled into contigs using Canu 2.1.1. Alignments between contigs and reference genomes were generated by MUMmer-nucmer 3.23 with default parameters and were used to create collinearity dot plot by Dot (https://github.com/dnanexus/dot).<br>For the stability of neochromosomes, after adapter removal, the low-quality reads were trimmed by Trimmomatic with parameters “SLIDINGWINDOW:5:20 LEADING:5 TRAILING:5 MINLEN:50”, and the remaining reads were mapped to reference genome with BWA-mem. |

After marking PCR repeat using GATK-MarkDuplicates, the SNPs were called by GATK- HaplotypeCaller and GATK- GenotypeGVCFs. Structural variations were called using DELLY.
We used Cutadapter software to remove adapters in raw RNA-seq data. HISAT2 and Picard were then used to accomplish the alignments of cleaned reads and remove PCR duplicates. Hereafter, Htseq-count software was employed to calculate read counts of each gene while intersection-nonempty option was set. The downstream statistical analysis was achieved by the DEseq2 package in R. For Spatial Analysis for Functional Enrichment (SAFE) analysis, the SAFE add-on with default parameters in cytoscape was used.
For Iso-seq data, circular Consensus Sequencing (CCS) reads were generated using SMRT-Link (version 8.0.0.80529), with the following modified parameters: "--min-passes 0 --min-length 50 --max-length 21000 --min-rq 0.75". We used Lima (version 1.10.0, commit SL-release-8.0.0) for Single Cell Full-Length Non-Concatemer (FLNC) reads detection. After FLNC detection, primer and polyA tail trimming, the remaining fraction of each isoform FLNC read was split into wild-type genome derived reads and neochromosome derived reads according to PCRtags using LAST software (https://gitlab.com/mcfrith/last), except the ptSYN10 libraries (alignment methods can distinguish the completely recoded genes on ptSYN10 from the wild-type ones). The neochromosome derived reads or all reads from ptSYN10 libraries were aligned to the designate reference genome with minimap2 (version 2.17-r974-dirty) in spliced alignment mode with parameters: "-ax splice -uf --secondary=no -C5". Reads with more than 95% identity and 30% coverage of a certain CDS are directly counted from paf files. To ensure the generation of transcripts with high accuracy, we use cDNA_Cupcake (https://github.com/Magdoll/cDNA_Cupcake) python script "collapse_isoforms_by_sam.py" to collapse redundant isoforms. The "--flnc-coverage" for minimum collapsed reads is set to 5 and the other parameters are set to default. After redundant isoforms collapsing, unique isoforms can be reported as GFF file. A homemade R script is used to illustrate the TSS, TES and transcription direction based on gggenes (https://github.com/wilkox/gggenes).
Raw metabolome data was converted into mzML format by ProteoWizard software. Peak extraction, peak alignment and retention time correction were respectively performed by XCMS program. The "SVR" method was used to correct the peak area. The peaks with detetion rate lower than 50% in each group of samples were discarded. After that, metabolic identification information was obtained by searching the laboratory's self-built database, integrated public database, AI database and metDNA. Data were loaded in R (www.r-project.org) and unsupervised principal component analysis (PCA) was performed by statistics function prcomp. The hierarchical cluster analysis (HCA) results of samples and metabolites were presented as heatmaps with dendrograms, while pearson correlation coefficients (PCC) between samples were caculated by the cor function in R and presented as only heatmaps. Both HCA and PCC were carried out by R package ComplexHeatmap.Identified metabolites were annotated using KEGG Compound database (http://www.kegg.jp/kegg/compound/) and annotated metabolites were then mapped to KEGG Pathway database (http://www.kegg.jp/kegg/pathway.html). Significantly enriched pathways are identified with a hypergeometric test's P-value for a given list of metabolites. For visualization of DMs in the metabolic network, the KEGG compound IDs of DMs (8 h) are mapped to iPATH3 yeast metabolism pathway map (https://pathways.embl.de/, species filter set to "sce") to generate dots. The uniprot ID of differentially expressed genes from transcriptomic data are also mapped to the identical map to generate lines.
Differential fitness scoring, the original images were in the format of .JPG with a resolution of 3168pixel x 4752pixel. To meet the resolution restriction of CellProfiler, the original images were unified compressed by 50% before the modified pipeline from the example on the website (https://cellprofiler.org/examples). To identify colonies effectively, we set the "Typical diameter of objects" parameter in the "Identify Primary Objects" step as 2 to 150, and manually checked the positions of the forced spots and the natural spots to avoid misidentification. The "Mean Intensity" value of the spots in the fourth gradient, where the wild-type strain did not reach the up limit of the "Mean Intensity" value, was used as a measure of cell growth for corresponding strains.
For FACS analysis, the software FlowJo was used for analysis and the fraction of cells at different stages was calculated with the Dean-Jett-Fox model.
For doubling time assay, the doubling time (D) of each strain were acquired using the GraphPad Prism 8 software.

For manuscripts utilizing custom algorithms or software that are central to the research but not yet described in published literature, software must be made available to editors and reviewers. We strongly encourage code deposition in a community repository (e.g. GitHub). See the Nature Portfolio guidelines for submitting code & software for further information.

## Data

Policy information about availability of data

All manuscripts must include a data availability statement. This statement should provide the following information, where applicable:

- Accession codes, unique identifiers, or web links for publicly available datasets
- A description of any restrictions on data availability
- For clinical datasets or third party data, please ensure that the statement adheres to our policy

Nanopore sequencing data, RNA-seq data and Iso-seq data have been deposited at SRA with bioproject number PRJNA883530 (https://www.ncbi.nlm.nih.gov/bioproject/PRJNA883530) and are publicly available as of the date of publication. Source data are provided with this paper.

## Research involving human participants, their data, or biological material

Policy information about studies with human participants or human data. See also policy information about sex, gender (identity/presentation), and sexual orientation and race, ethnicity and racism.

| | |
|---|---|
| Reporting on sex and gender | N/A |
| Reporting on race, ethnicity, or other socially relevant groupings | N/A |
| Population characteristics | N/A |
| Recruitment | N/A |
| Ethics oversight | N/A |

Note that full information on the approval of the study protocol must also be provided in the manuscript.

# Field-specific reporting

Please select the one below that is the best fit for your research. If you are not sure, read the appropriate sections before making your selection.

[x] Life sciences [ ] Behavioural & social sciences [ ] Ecological, evolutionary & environmental sciences

For a reference copy of the document with all sections, see nature.com/documents/nr-reporting-summary-flat.pdf

# Life sciences study design

All studies must disclose on these points even when the disclosure is negative.

| | |
|---|---|
| Sample size | The sample size of each experiment is provided in the figure legends in the main manuscript and supplementary files. For RNA-seq, cell imaging, blotting, phenotype analysis, metabolic analysis and FACS analysis, samples were prepared in at least three biological replicates. For the stability analysis of the neo-chromosomes, 20 colonies were used for PCRtag analysis and 3 colonies were used for WGS analysis. For Iso-seq, two independent biological samples for each genotype were sequenced and the detail results in one sample were used to show the pattern. |
| Data exclusions | No data was excluded from analysis, except for necessary data quality control step for RNA-seq data processing. |
| Replication | All experiments were performed at least three times, except the Iso-seq experiments with two biological repeats. All attempts at replication were successful. |
| Randomization | Randomization is not relevant because no group allocation was involved in this study. |
| Blinding | Blinding is not relevant because no group allocation was involved in this study. |

# Reporting for specific materials, systems and methods

We require information from authors about some types of materials, experimental systems and methods used in many studies. Here, indicate whether each material, system or method listed is relevant to your study. If you are not sure if a list item applies to your research, read the appropriate section before selecting a response.

## Materials & experimental systems

| n/a | Involved in the study | |
|---|---|---|
| [x] | [ ] | Antibodies |
| [x] | [ ] | Eukaryotic cell lines |
| [x] | [ ] | Palaeontology and archaeology |
| [x] | [ ] | Animals and other organisms |
| [x] | [ ] | Clinical data |
| [x] | [ ] | Dual use research of concern |
| [x] | [ ] | Plants |

## Methods

| n/a | Involved in the study | |
|---|---|---|
| [x] | [ ] | ChIP-seq |
| [ ] | [x] | Flow cytometry |
| [x] | [ ] | MRI-based neuroimaging |

## Plants

| | |
|---|---|
| Seed stocks | *Report on the source of all seed stocks or other plant material used. If applicable, state the seed stock centre and catalogue number. If plant specimens were collected from the field, describe the collection location, date and sampling procedures.* |
| Novel plant genotypes | *Describe the methods by which all novel plant genotypes were produced. This includes those generated by transgenic approaches, gene editing, chemical/radiation-based mutagenesis and hybridization. For transgenic lines, describe the transformation method, the number of independent lines analyzed and the generation upon which experiments were performed. For gene-edited lines, describe the editor used, the endogenous sequence targeted for editing, the targeting guide RNA sequence (if applicable) and how the editor was applied.* |
| Authentication | *Describe any authentication procedures for each seed stock used or novel genotype generated. Describe any experiments used to assess the effect of a mutation and, where applicable, how potential secondary effects (e.g. second site T-DNA insertions, mosiacism, off-target gene editing) were examined.* |

# Flow Cytometry

## Plots

Confirm that:

- [x] The axis labels state the marker and fluorochrome used (e.g. CD4-FITC).
- [x] The axis scales are clearly visible. Include numbers along axes only for bottom left plot of group (a 'group' is an analysis of identical markers).
- [ ] All plots are contour plots with outliers or pseudocolor plots.
- [x] A numerical value for number of cells or percentage (with statistics) is provided.

## Methodology

| | |
|---|---|
| Sample preparation | Samples were selected at corresponding time points and cells were fixed with 70% ethanol overnight at 4°C. Cells were resuspended in 50mM sodium citrate (pH 7.0) and briefly sonicated on ice. Cells were resuspended in 50 mM sodium citrate (pH 7.0) and added with RnaseA (0.25 mg/mL) for incubation at 37°C. Cells were washed with 50mM sodium citrate (pH 7.0) and resuspended in 50mM sodium citrate (pH 7.0) containing propidium iodide (16 µg/mL). The cells were incubated at room temperature for at least 1 hour. |
| Instrument | Samples were proceeded with BD FACS Celesta for measurement. |
| Software | The software FlowJo was used for analysis and the fraction of cells at different stages was calculated with the Dean-Jett-Fox model. |
| Cell population abundance | For each sample, 20000 events were recorded. After the removement of potential cellular debris or adhesion of cells, more then 17000 original events were selected for analysis of each sample. |
| Gating strategy | Due to the difference of cell size between WT and ySYN24, the majority of cell population (>85%) was selected manually based on the distribution of events in the view of SSC-A/FSC-A. |

- [ ] Tick this box to confirm that a figure exemplifying the gating strategy is provided in the Supplementary Information.
