# Supplementary material for: Building a eukaryotic chromosome arm by de novo design and synthesis
Source: Nat Commun. 2023 Nov 30;14:7886. doi: 10.1038/s41467-023-43531-5 (PMC10689750; doi:10.1038/s41467-023-43531-5)
Supplement: Supplementary file 9 — Peer Review File [file 41467_2023_43531_MOESM9_ESM.pdf]

nature portfolio

Peer Review File

Building a eukaryotic chromosome arm by de novo design and synthesis

Open Access This file is licensed under a Creative Commons Attribution 4.0 International License, which permits use, sharing, adaptation, distribution and reproduction in any medium or format, as long as you give appropriate credit to the original author(s) and the source, provide a link to the Creative Commons license, and indicate if changes were made. In the cases where the authors are anonymous, such as is the case for the reports of anonymous peer reviewers, author attribution should be to 'Anonymous Referee' followed by a clear attribution to the source work. The images or other third party material in this file are included in the article's Creative Commons license, unless indicated otherwise in a credit line to the material. If material is not included in the article's Creative Commons license and your intended use is not permitted by statutory regulation or exceeds the permitted use, you will need to obtain permission directly from the copyright holder. To view a copy of this license, visit http://creativecommons.org/licenses/by/4.0/.

Reviewers' Comments:

Reviewer #1:
Remarks to the Author:
High level summary: very good work.

Firstly, the authors attempted to replace the left arm of yeast chromosome XII with a pre-validated principle of using linear artificial chromosome harboring a significantly reduced set of genes. The initial attempt of using just 10 previously defined individually "essential" genes failed to yield viable clones, which is hardly surprising as scientists now appreciate the importance and intricacy of synthetic combinatorial lethal and the incomplete human understanding of gene networks and functions. The fundamental challenge is how to re-substitute in "non-essential" genes to rescue the lethality. Following a linear trajectory, the authors then systematically complemented the 10 "essential genes" with one of 49 "non-essential" genes. None of the single re-substitution worked.

What the authors demonstrated next is a good intelligence contribution. After failure to rescue non-growth phenotype with a single re-substitution, the authors "linearly" moved on with double re-substitutions of two "non-essential" genes. But here presents a fundamental mathematical challenge and impracticality of exhausting all C492 = 1,176 combinations. This challenge is further exponentially amplified as the number of "non-essential" genes needed to be added back in increase. Hence a practical, scalable solution is needed.

The authors ingeniously hypothesized using GGI as an indication to identify a small subset of genes (gene's GGI over certain thresholds) to be added back in. And it worked! The authors not only demonstrated the feasibility of identifying 2 additional genes to re-constitute in addition to the initial 10 to sustain cell viability with just 12 genes, they went further to further improve the growth phenotype with the identification of another 13 genes to a total of 25.

They then went even further to recode these genes with a very aggressive recoding scheme coupled with synthetic transcription control elements. They devised a very smart selection scheme to test and validate variable combinations of the coding sequences and control elements and successfully identified working combinations for 24 of the 25 genes, with one not tested due to failed assembly.

They then showed that the synthetic linear chromosome can function by itself, despite with significant growth defects, which is expected considering the importance of codon choices.

It is a great work!

Would recommend for publication.

However, some revision can definitely make the paper clearer to the readers.

In particular:

1. A more thorough description regarding why the left arm of yeast chromosome XII is chosen as the testing bed would be very helpful. Currently it seems very random. For whatever criteria, it would be better to show the comparison of this chosen region to the rest of yeast genome.

2. 150-151 Why the CYC1 reporter is chosen?

3. 413-414 Understand it is given as a reference, but it would still be ideal to briefly discuss the design principles of the recoding scheme and how GeneDesign generates it. A detailed information of which codon is used for which amino acid would be needed here. Also, ideally explanation as why the codon is chosen out of other synonymous codons.

4. A sequence list of promoters and terminators would be needed.

5. 470-472 What happened to UIB4 on ptSYN24 in comparison to ptWT25? Please clarify? Is the wildtype copy of UIB4 used in ptSYN24? Or is the UIB4 simply not included in ptSYN24?

In general, again, great work!

Reviewer #2:
Remarks to the Author:
In this manuscript, the authors report the minimization of yeast chromosome XII left arm (chrXIIL, 150 kb) by introduction of an episomal essential gene array (10 genes) and chromosomal truncation, and the impact of these operations is analyzed in details.

However, the research ideas and technical strategy of this manuscript are very similar to two previously their published papers. The authors have already done a similar study on compacting the same chromosome arm chrXIIL, although with a slight difference in the way of chromosomal deletions (Luo et al., Genome Biol., 2021). The codon compression of 10 essential genes of chrXIIL has also been published (Liang et al., iScience, 2022). It seems like this manuscript is an upgraded version of these two articles. Furthermore, the detailed assessment of biological impacts on chrXIIL deletions in this study doesn’t seem to have any significant new findings. Therefore, both the scientific significance and novelty of this work are not clear.

Reviewer #3:
Remarks to the Author:
This research was conducted as one of the pilot studies within the context of the international collaboration project, Sc3.0. The team's objective was to explore genome plasticity. They did this by replacing the native left arm of chromosome XII with a linear artificial chromosome. This new chromosome was designed to carry small clusters of genes that underwent various substantial modifications. These included the identification of minimal gene sets required for cell viability and the remediation of fitness defects. Additionally, the project involved recoding of promoters, terminators, and open-reading frames using a 'one-amino-acid-one codon' strategy. Despite the high-risk nature of the project, it promises high rewards, having already produced uniquely insightful results.

On the other hand, given the intricate nature of the project, it is essential to provide a clearer interpretation of certain results, particularly for readers who may not be familiar with the preceding Sc2.0 project.

1. Line 76: “To facilitate genome reduction, we recently developed an iterative SCRaMbLE-based genome compaction (SGC) strategy, which allowed us to remove about 40% of synXIIL randomly while the cells remained viable at 30°C in rich medium.”

- "Removing about 40% of synXIIL randomly" may appear ambiguous without prior knowledge of the previous publication. Removing 40% of a chromosome in a random manner is certainly not feasible due to the essentiality of certain genes. While SCRaMbLE is conducted randomly to introduce deletions, insertions, inversions, and other changes, it does not imply that 40% of a chromosome can be arbitrarily removed.

2. Line 168: “Interestingly, we found that the telomeric TG repeats, in both neochromosomes, significantly expanded (Extended Data Fig.2h-i), indicating the TeSS end grew a new telomere successfully, as designed.”

- The phrase "as designed" can be confusing in this context as it may imply that the authors were already aware that the TeSS end would grow.

3. I have two inquiries regarding Figure 1j, 1k, and 1m, and I would appreciate it if the authors could provide some speculations:

(a) Why did ptWT10U exhibit a higher copy number compared to ptWT10?

(b) Following normalization by His3, why did YLL031C display a significantly decreased transcription level in ptWT10U-10KO compared to ptWT10-10KO?

4. Line 244: “Six regions flanking essential genes not fully deleted by SCRaMbLE in our previous study were knocked out individually (Extended Data Fig.5a).”

- I find the term "not fully deleted" to be confusing. Did the authors intend to convey that SCRaMbLE only removed certain portions of the six regions but not the entire regions?

5. Regarding Extended Data Figure 5 (Line 247), I have the following questions:

(a) Why were only two genes labeled in the figure?

(b) What was the reason for region one needing to overlap with the ORF of YLL002W?

6. Line 251, Figure 2a: “In this method, a fragment containing a universal telomere, a marker gene and a homologous region is transformed into yeast to create a new telomere at the left end of chrXII by homologous recombination.”

- This design is unconventional since the homology arm is present on only one side of the marker. Is this a single cross-over mechanism? It seems that this design is feasible due to the presence of a telomere on the other side of the marker, correct?

7. Line 371: “Seven out of the eight genes were also retained in ZLY348, the strain with compacted chrXIIL but retaining wild type-like growth.”

- Providing additional information about the compacted chrXIIL would be beneficial and add value to the understanding of the topic.

8. Line 338: “YLL013C (PUF3), the gene encoding a mitochondrial outer surface protein with over 2000 putative mRNA targets, was also included.”

- This sentence appears unclear. Are the authors suggesting that approximately 2000 putative mRNAs will be transported into the mitochondria for translation?

9. A typo in Line 376: “adn” should be “and”.

10. Regarding Figure 5a, top row: After the "plasmid isolation" step, why does the strain transition from a haploid feature to a heterozygous state (WT+eCDS deletion)?

11. Several techniques employed in this project may not be familiar to a general audience. To enhance readability, it would be helpful to provide a couple of clarifying sentences upon their first mention.

(a) Line 193: Isoform-sequencing - It would be beneficial to explain what information can be obtained through this technique.

(b) Line 274: Genetic interaction with a target gene (GGI) - It would be valuable to elaborate on how these interactions were initially measured.

(c) Line 310: MMS and rapamycin-induced chemical stress - Providing information about which pathways are affected by MMS and rapamycin-induced chemical stress would enhance understanding.

(d) Line 361: Principal component analysis - Since this technique may not be widely known, it

would be advantageous to provide a brief description of what insights can be derived from principal component analysis.

Reviewer #1 (Remarks to the Author):

High level summary: very good work.

Firstly, the authors attempted to replace the left arm of yeast chromosome XII with a pre-validated principle of using linear artificial chromosome harboring a significantly reduced set of genes. The initial attempt of using just 10 previously defined individually "essential" genes failed to yield viable clones, which is hardly surprising as scientists now appreciate the importance and intricacy of synthetic combinatorial lethal and the incomplete human understanding of gene networks and functions. The fundamental challenge is how to re-substitute in "non-essential" genes to rescue the lethality. Following a linear trajectory, the authors then systematically complemented the 10 "essential genes" with one of 49 "non-essential" genes. None of the single re-substitution worked.

What the authors demonstrated next is a good intelligence contribution. After failure to rescue non-growth phenotype with a single re-substitution, the authors "linearly" moved on with double re-substitutions of two "non-essential" genes. But here presents a fundamental mathematical challenge and impracticality of exhausting all C492 = 1,176 combinations. This challenge is further exponentially amplified as the number of "non-essential" genes needed to be added back in increase. Hence a practical, scalable solution is needed.

The authors ingeniously hypothesized using GGI as an indication to identify a small subset of genes (gene's GGI over certain thresholds) to be added back in. And it worked! The authors not only demonstrated the feasibility of identifying 2 additional genes to re-constitute in addition to the initial 10 to sustain cell viability with just 12 genes, they went further to further improve the growth phenotype with the identification of another 13 genes to a total of 25.

They then went even further to recode these genes with a very aggressive recoding scheme coupled with synthetic transcription control elements. They devised a very smart selection scheme to test and validate variable combinations of the coding sequences and control elements and successfully identified working combinations for 24 of the 25 genes, with one not tested due to failed assembly.

They then showed that the synthetic linear chromosome can function by itself, despite with significant growth defects, which is expected considering the importance of codon choices.

It is a great work!

Would recommend for publication.

However, some revision can definitely make the paper clearer to the readers.

In particular:

1. A more thorough description regarding why the left arm of yeast chromosome XII is chosen as the testing bed would be very helpful. Currently it seems very random. For whatever criteria, it would be better to show the comparison of this chosen region to the rest of yeast genome.

Response: We thank the reviewer for the suggestion. This work is based on our previous study on the construction of a synthetic chromosome XII or *SynXII* (Zhang et al., Science, 2017), and initiated at the early stage during the construction of *SynXII*. Therefore, we just used the materials at hand to start this work.

As suggested, we compared the left arm of chromosome XII to the rest of yeast genome as following:
1) Yeast chromosome arms vary in length, ranging from 78.6 kb to about 1 Mb. The chosen *chrXIIL* is about 150kb in length, the seventh shortest chromosome arm in yeast, and twice the length of the shortest arm.
2) We compared the protein length distribution of *chrXIIL* to that of the entire yeast genome. The length of proteins encoded by this chromosome arm showed no significant difference to that of genome-wide genes (Supplementary Fig.1a).
3) We also compared the functional complexity of the genes on *chrXIIL* to the ones on other arms. The average number of directly evidenced gene ontology (GO) terms per gene on *chrXIIL* was slightly higher than other chromosome arms (Supplementary Fig.1b).

Accordingly, we have revised the texts as follows:

- The left arm of *chrXII* in S. cerevisiae (*chrXIIL*) is 150,827 bp long, ranking as the seventh shortest yeast arm. It contains 74 genes (62 protein coding genes, 9 dubious genes, 2 pseudogenes, 1 tRNA coding gene), 3 autonomously replicating sequences and 1 Ty1 LTR (Saccharomyces Genome Database, https://www.yeastgenome.org/). Among these genes, 10 are defined as essential based on the phenotype of individual knockout mutations and their ability to support spore viability in a heterozygous diploid. The length of proteins encoded by genes on *chrXIIL*, showed a similar distribution pattern to that of genome-wide genes (Supplementary Fig.1a). The average number of directly evidenced gene ontology (GO) terms per gene on chrXIIL was slightly higher than other chromosome arms, suggesting higher functional complexity to be considered during reconstruction (Supplementary Fig.1b).

2. 150-151 Why the CYC1 reporter is chosen?

Response: *YLL035W* and *YLL036C* share a bi-directional promoter. However, according to the previous study (Ref.21 in texts), the shared 323-bp intergenic region is not able to sustain the function of *YLL036C* and the coding sequence of *YLL035W* contains essential components of *YLL036W*'s promoter. In the same study (Ref.21 in texts), the *CYC1*

promoter and terminator were used for the expression of synthetic versions of *YLL035W* and *YLL036C*, and the resulted strains did not show obvious growth defects, suggesting the *CYC1* promoter could drive the expression of the two genes to support cell viability. To keep both genes functional and under similar regulation, we used the *CYC1* promoter to drive the expression of *YLL035W* and *YLL036C*.

To make it clear, we have revised the texts as follows:

- In addition, since *YLL035W* and *YLL036C* share a bi-directional promoter and the intergenic region is unable to sustain the function of *YLL036C*[21], we chose the reported *CYC1* promoter to achieve functional expression of both genes and maintain consistent regulation in ptWT10U.

3. 413-414 Understand it is given as a reference, but it would still be ideal to briefly discuss the design principles of the recoding scheme and how GeneDesign generates it. A detailed information of which codon is used for which amino acid would be needed here. Also, ideally explanation as why the codon is chosen out of other synonymous codons.

Response: Thanks for the suggestions. The information regarding which codon is utilized for each amino acid has been incorporated in Supplementary Fig.8a.

We have revised the texts as follows:

- For coding sequences (CDS), the optimized DNA sequences were generated using GeneDesign software as before [21,39], which employs a radical codon compression scheme, in which only one optimal codon is used for each amino acid (Supplementary Fig.8a). And the optimal codon for each amino acid was defined by the highest relative synonymous codon usage (RSCU) value in highly expressed genes in yeast genome[21,39].

4. A sequence list of promoters and terminators would be needed.

Response: Thanks for the suggestions. We have added the sequence list for 44 short synthetic promoters and 28 short synthetic terminators in Supplementary Table 3.

5. 470-472 What happened to UIB4 on ptSYN24 in comparison to ptWT25? Please clarify? Is the wildtype copy of UIB4 used in ptSYN24? Or is the UIB4 simply not included in ptSYN24?

Response: We failed to assemble the recoded TUs of *UBI4*, potentially due to its repetitive nature since it encodes 5 head-to-tail ubiquitin repeats within CDS. Therefore, the *UIB4* was not included in ptSYN24.

We have modified the texts as follows:

- Next, based on what we learned above using native genes, we constructed the ptSYN24 neochromosome (Fig. 6a), which contains 24 recoded TUs from ptWT25 without *UBI4*.

Reviewer #2 (Remarks to the Author):

In this manuscript, the authors report the minimization of yeast chromosome XII left arm (chrXIIL, 150 kb) by introduction of an episomal essential gene array (10 genes) and chromosomal truncation, and the impact of these operations is analyzed in details.

However, the research ideas and technical strategy of this manuscript are very similar to two previously their published papers. The authors have already done a similar study on compacting the same chromosome arm chrXIIL, although with a slight difference in the way of chromosomal deletions (Luo et al., Genome Biol., 2021). The codon compression of 10 essential genes of chrXIIL has also been published (Liang et al., iScience, 2022). It seems like this manuscript is an upgraded version of these two articles. Furthermore, the detailed assessment of biological impacts on chrXIIL deletions in this study doesn't seem to have any significant new findings. Therefore, both the scientific significance and novelty of this work are not clear.

Response: We appreciate the reviewer's feedback. We highlight the scientific significance and novelty of this work as follows:

- Firstly, there are notable differences between the research ideas and technical strategy to simplify the *chrXIIL* in this study from that of our previous report in Genome Biology (Luo et al., Genome Biol., 2021). In the previous study, the SCRaMbLE-based genome compaction (SGC) method was developed to enable random deletion of chromosome regions. We solely relied on analyzing the sequences in survival strains to determine which regions in the chromosome are dispensable. However, due to the limited number of strains to be sequenced, it is not easy, if not impossible, to test the essentiality of all chromosome regions. Therefore, SGC gave us a way to quickly slim down the genome. In this study, we took a step further and tried to identify the minimal sequences/genes to substitute the entire arm. Therefore, we systematically examined the essentiality of sequences in the left arm of chrXII using CRISPR/Cas9 technology and a chromosome truncation method. Furthermore, to obtain the minimal sequences/genes, we not only tried to delete the native sequences, but also constructed an essential set of sequences/genes from scratch using cell viability as a gauge. This study led us to uncover a new fact, i.e., that only twelve genes among over 70 genes in the left arm of chromosome XII are required for cell viability. This finding has also prompted us to speculate that a similar situation may exist in other chromosomes, suggesting that the redundancy of the yeast genome is unparalleled.
- Secondly, as the reviewer pointed out that the codon compression of 10 individual essential genes of *chrXIIL* has been published in the iScience paper. However, the purpose of this study for using the recoded CDS is to test whether a native chromosome arm could be replaced totally using artificial sequences (the sequences do not exist in the nature). Therefore, we not only recoded the CDS, but also employed synthetic transcription control elements, and developed an efficient selection scheme to identify functional combinations for these genes. Our results indicate that it is

possible to redesign a neochromosome using totally artificial sequences to substitute the native chromosome arm, which has never been achieved before.

- Thirdly, when picking genes required for cell viability, it is obvious to include the essential genes. However, besides these essential genes, there is no guidance on how to choose the other “less important” genes. In this study, we demonstrated that GGI could be used as a good indication. By including different sets of genes based on GGI, we found that 10 essential genes plus only two non-essential genes form currently the minimal gene set sufficient to replace *chrXIIL* for cell viability. Subsequently, we identified 13 more genes for robust fitness without numerous workloads.

Together, we hope these facts could convince the reviewer that this manuscript not just an upgraded version of the previous studies.

Reviewer #3 (Remarks to the Author):

This research was conducted as one of the pilot studies within the context of the international collaboration project, Sc3.0. The team's objective was to explore genome plasticity. They did this by replacing the native left arm of chromosome XII with a linear artificial chromosome. This new chromosome was designed to carry small clusters of genes that underwent various substantial modifications. These included the identification of minimal gene sets required for cell viability and the remediation of fitness defects. Additionally, the project involved recoding of promoters, terminators, and open-reading frames using a 'one-amino-acid-one codon' strategy. Despite the high-risk nature of the project, it promises high rewards, having already produced uniquely insightful results.

On the other hand, given the intricate nature of the project, it is essential to provide a clearer interpretation of certain results, particularly for readers who may not be familiar with the preceding Sc2.0 project.

1. Line 76: "To facilitate genome reduction, we recently developed an iterative SCRaMbLE-based genome compaction (SGC) strategy, which allowed us to remove about 40% of synXIIL randomly while the cells remained viable at 30°C in rich medium."

- "Removing about 40% of synXIIL randomly" may appear ambiguous without prior knowledge of the previous publication. Removing 40% of a chromosome in a random manner is certainly not feasible due to the essentiality of certain genes. While SCRaMbLE is conducted randomly to introduce deletions, insertions, inversions, and other changes, it does not imply that 40% of a chromosome can be arbitrarily removed.

Response: Thanks for pointing this out.

We have changed the texts as follows:

- To facilitate genome reduction, we recently developed an iterative SCRaMbLE-based genome compaction (SGC) strategy, which allowed us to remove about 40% of *synXIIL* while the cells remained viable at 30°C in rich medium[12].

2. Line 168: "Interestingly, we found that the telomeric TG repeats, in both neochromosomes, significantly expanded (Extended Data Fig.2h-i), indicating the TeSS end grew a new telomere successfully, as designed."

- The phrase "as designed" can be confusing in this context as it may imply that the authors were already aware that the TeSS end would grow.

Response: We agree with the reviewer that the phrase "as designed" is confusing.

Therefore, we have deleted the phrase and revised the texts as follows:

- Interestingly, we found that the telomeric TG repeats, in both neochromosomes, significantly expanded (Supplementary Fig.2h-i), indicating the TeSS end grew a new telomere successfully[2,27].

3. I have two inquiries regarding Figure 1j, 1k, and 1m, and I would appreciate it if the authors could provide some speculations:

(a) Why did ptWT10U exhibit a higher copy number compared to ptWT10?

Response: This might be a misunderstanding of the data. We employed a two-tailed t-test to compare the copy numbers of ptWT10 and ptWT10U, and no statistically significant differences were observed (P>0.1). Therefore, our conclusion is that the copy number of the neochromosomes varies among different clones with the same genotype, and there is no significant difference between the copy numbers of ptWT10U and ptWT10.

(b) Following normalization by His3, why did YLL031C display a significantly decreased transcription level in ptWT10U-10KO compared to ptWT10-10KO?

Response: We are also curious about it. There are several possibilities: 1) The major distinction between ptWT10-10KO and ptWT10U-10KO is the alteration of the regulatory sequences of *YLL035W/GRC3* and *YLL036C/PRP19*, which could also potentially impact the expression of *YLL031C*. 2) Among the specific differentially expressed genes in ptWT10U-10KO, the two repressive transcription factors, *YDR207C/UME6* and *YDR259C*/YAP6, were down-regulated（Supplementary Table 2). These two genes have been reported to be regulators of *YLL031C* （MacIsaac KD, et al. 2006, BMC Bioinformatics 7:113; Venters BJ, et al. 2011. Mol Cell 41(4):480-92). Another specific differentially expressed gene in ptWT10U-10KO, *SUR1/YPL057C,* exhibits genetic interactions with *YLL035W*, *YLL036C*, *UME6* and *YLL031C* (SGD database). Above information suggests that the altered expression of *YLL035W* and *YLL036C* in ptWT10U-10KO may indirectly affect the expression of *YLL031C*. 3) In comparison to BY4742, the expression of *YLL031C* in both ptWT10-10KO and ptWT10U-10KO was ~$2^{4.5}$-fold higher, suggesting the presence of a potential feed-back mechanism to keep the expression of *YLL031C* below a certain threshold.
Together, we speculate that the expression of *YLL031C* is likely influenced by multiple factors.

4. Line 244: “Six regions flanking essential genes not fully deleted by SCRaMbLE in our previous study were knocked out individually (Extended Data Fig.5a).”

- I find the term "not fully deleted" to be confusing. Did the authors intend to convey that SCRaMbLE only removed certain portions of the six regions but not the entire regions?

Response: Thanks for pointing this out. Yes, SCRaMbLE only deleted certain portions of the six regions in the strain ZLY349 by removing the gene contents between two loxPsym sites in our previous research (Luo et al., Genome Biol., 2021). ZLY349 is the viable strain with the most sequence deleted (58% of *synXIIL* sequences) in that study and chosen for visualization of removed parts (dashed lines, Supplementary Fig.5a). Almost all the sequences flanking essential genes before HR II were removed in ZLY349, while the six regions following HR II were either partially deleted or retained. So, our analysis focused on examining the essentiality of these six regions.

We have modified the texts as follows:

- However, further compaction attempts using the same method failed, which presumably may be partially due to the extremely slow growth of the final strain ZLY349[12]. To probe the minimal gene set to support cell viability, two additional strategies were carried out here.
  At first, we systematically examined the essentiality of sequences in the left arm of *chrXII* using CRISPR/Cas9 technology. The six regions flanking essential genes, which were either partially deleted or retained in ZLY349[12], were knocked out individually (Supplementary Fig.5a).

5. Regarding Extended Data Figure 5 (Line 247), I have the following questions:

(a) Why were only two genes labeled in the figure?

Response: *YLL002W* and *YLL006W* are the two non-essential genes added to the neochromosome to maintain cell viability. They were labeled in the figure to help understanding the phenotypes of strains generated through various deletion methods. For instance, *YLL002W* was one of the two genes disrupted in the Δ1 strain, which showed severe growth defects. Both genes were located in region IV, between HR III and HR IV. These results also suggest the importance of these genes.

(b) What was the reason for region one needing to overlap with the ORF of YLL002W?

Response: In the Δ1 strain, we wanted to delete all the non-essential genes before *YLL003W* on the left arm of chromosome XII. To reduce the potential impact of deleting non-essential genes on neighboring essential genes, we preserved approximately 500 bp of upstream sequences and 300 bp of downstream sequences of essential genes. The intergenic region between *YLL003W* and *YLL002W* is approximately 250 bp, so that about 50bp sequences in the N terminal of *YLL002W* were retained.

6. Line 251, Figure 2a: "In this method, a fragment containing a universal telomere, a marker gene and a homologous region is transformed into yeast to create a new telomere at the left end of chrXII by homologous recombination."

- This design is unconventional since the homology arm is present on only one side of the marker. Is this a single cross-over mechanism? It seems that this design is feasible due to the presence of a telomere on the other side of the marker, correct?

Response: Yes, you are right! This is a single cross-over mechanism. And this design is feasible due to the presence of a telomere on the other side of the marker.

7. Line 371: “Seven out of the eight genes were also retained in ZLY348, the strain with compacted chrXIIL but retaining wild type-like growth.”

- Providing additional information about the compacted *chrXIIL* would be beneficial and add value to the understanding of the topic.

Response: Thanks for the suggestions.

We have added more information about the compacted arm in ZLY348 in the revised texts as follows:

➢ Seven out of the eight genes were also retained in ZLY348, the strain with a compacted *chrXIIL* (~40% removal of *synXIIL* sequences) but retaining wild type-like growth on YPD at 30°C[12].

8. Line 338: “YLL013C (PUF3), the gene encoding a mitochondrial outer surface protein with over 2000 putative mRNA targets, was also included.”

- This sentence appears unclear. Are the authors suggesting that approximately 2000 putative mRNAs will be transported into the mitochondria for translation?

Response: Thanks for pointing this out. Puf3p is an mRNA-bind protein that plays important roles in deadenylation-dependent mRNA decay. This protein is also involved in mitochondrion organization and localization. It is localized to the cytoplasm and the cytoplasmic side of the mitochondrial outer membrane. We are not suggesting that approximately 2000 putative mRNAs (nearly a third of the yeast transcriptome) will be transported into the mitochondria for translation.

We have modified the texts as follows:

➢ *YLL013C (PUF3)*, a gene that encodes an mRNA binding protein involved in mRNA decay processes and known to have over 2000 putative mRNA targets[36], was also included (Supplementary Fig.6f).

9. A typo in Line 376: “adn” should be “and”.

Response: This has been corrected.

10. Regarding Figure 5a, top row: After the "plasmid isolation" step, why does the strain transition from a haploid feature to a heterozygous state (WT+eCDS deletion)?

Response: To validate the functionality of the candidate plasmids for essential genes, two methods can be employed: the 5-FOA shuffling assay using the corresponding haploid deletion mutant or the standard tetrad-based analysis with heterozygous strains. In this study, we selected the 5-FOA shuffling assay for screening and chose the tetrad dissection analysis for individual verification in a clean background.

11. Several techniques employed in this project may not be familiar to a general audience. To enhance readability, it would be helpful to provide a couple of clarifying sentences upon their first mention.

(a) Line 193: Isoform-sequencing - It would be beneficial to explain what information can be obtained through this technique.

Response: Thanks for the suggestions.

We have modified the texts as follows:

- Therefore, we examined whether there were any abnormalities in transcription initiation and termination sites of each gene in the two neochromosomes using the isoform-sequencing (Iso-seq) method, a high-throughput method to identify all full-length transcripts within a cell. From the Iso-seq results of BY4742 containing either ptWT10 or ptWT10U, the full-length transcripts of nine essential genes on the neochromosomes, were identified using specific PCRTags (except for *YLL050C*, which is too short to contain PCRTags)[30].

(b) Line 274: Genetic interaction with a target gene (GGI) - It would be valuable to elaborate on how these interactions were initially measured.

Response: Thanks for the suggestions.

We have modified the texts as follows:

- Thus, we tried to add two genes. Genetic interactions are quantified by measuring phenotypes of single and double mutants and calculating an interaction factor that reflects any deviation from the expected combined effect of the two single mutants[33]. To date, the investigations of genetic interaction have been extensively conducted in budding yeast, using genome-wide yeast mutant collections and automated colony size-scoring methodology[33]. It has been reported that essential genes participate in more genetic interactions than non-essential genes, raising the hypothesis that the

higher importance of the gene, the more genetic interactions may exist. Therefore, we used the number of genes that showed genetic interaction with a target gene (GGI) as a quantitative indicator of genetic interactions.

(c) Line 310: MMS and rapamycin-induced chemical stress - Providing information about which pathways are affected by MMS and rapamycin-induced chemical stress would enhance understanding.

Response: Thanks for the suggestions.

We have modified the texts as follows:

- As shown in Fig.3b, introducing the six genes could obviously improve the growth of cells, not only on rich medium, but also under the chemical stresses (DNA damaging agent MMS and mTOR inhibitor rapamycin).

(d) Line 361: Principal component analysis - Since this technique may not be widely known, it would be advantageous to provide a brief description of what insights can be derived from principal component analysis.

Response: Thanks for the suggestions.

We have described the principal component analysis in our revised text as follows:

- Principal component analysis (PCA) has been widely used as a multivariate method in metabolomics analysis, which is a key tool to identify patterns and outliers in the metabolomics datasets[37]. PCA of the metabolites composition among the three strains showed that the first principal component (PC1) versus PC2 accounted for over 49% of the total variation (Fig. 4a), which revealed a shift in metabolite profiles over time.

Reviewers' Comments:

Reviewer #1:
Remarks to the Author:
I am happy to see the authors explained in details the raised questions. I feel the manuscript is worthy of publication at its current status. Congratulations!

Reviewer #2:
Remarks to the Author:
No further questions.

Reviewer #3:
Remarks to the Author:
I greatly appreciate the authors' efforts in addressing the majority of my inquiries and believe the article is ready for acceptance.

Reviewer #1 (Remarks to the Author):

I am happy to see the authors explained in details the raised questions. I feel the manuscript is worthy of publication at its current status. Congratulations!

Response: We thank the reviewer for the comments.

Reviewer #2 (Remarks to the Author):

No further questions.

Response: We thank the reviewer for the comments.

Reviewer #3 (Remarks to the Author):

I greatly appreciate the authors' efforts in addressing the majority of my inquiries and believe the article is ready for acceptance.

Response: We thank the reviewer for the comments.
